# Characterization of Three Novel Virulent *Aeromonas* Phages Provides Insights into the Diversity of the *Autographiviridae* Family

**DOI:** 10.3390/v14051016

**Published:** 2022-05-10

**Authors:** Katarzyna Bujak, Przemyslaw Decewicz, Michal Kitowicz, Monika Radlinska

**Affiliations:** Department of Environmental Microbiology and Biotechnology, Faculty of Biology, Institute of Microbiology, University of Warsaw, Miecznikowa 1, 02-096 Warsaw, Poland; k.bujak@uw.edu.pl (K.B.); p.decewicz@uw.edu.pl (P.D.); m.kitowicz@student.uw.edu.pl (M.K.)

**Keywords:** bacteriophage, *Aeromonas*, *Autographiviridae*, cold-active, extreme environment, comparative genomics, SP6-like, phiKMV-like, RNA polymerase, tail fiber

## Abstract

In this study, we isolated and characterized three novel virulent *Autographiviridae* bacteriophages, vB_AspA_Bolek, vB_AspA_Lolek, and vB_AspA_Tola, which infect different *Aeromonas* strains. These three host–pathogen pairs were derived from the same sampling location—the arsenic-containing microbial mats of the Zloty Stok gold mine. Functional analysis showed they are psychrotolerant (4–25 °C), albeit with a much wider temperature range of propagation for the hosts (≤37 °C). Comparative genomic analyses revealed a high nucleotide and amino acid sequence similarity of vB_AspA_Bolek and vB_AspA_Lolek, with significant differences exclusively in the C-terminal region of their tail fibers, which might explain their host range discrimination. The protein-based phage network, together with a phylogenetic analysis of the marker proteins, allowed us to assign vB_AspA_Bolek and vB_AspA_Lolek to the *Beijerinckvirinae* and vB_AspA_Tola to the *Colwellvirinae* subfamilies, but as three novel species, due to their low nucleotide sequence coverage and identity with other known phage genomes. Global comparative analysis showed that the studied phages are also markedly different from most of the 24 *Aeromonas* autographiviruses known so far. Finally, this study provides in-depth insight into the diversity of the *Autographiviridae* phages and reveals genomic similarities between selected groups of this family as well as between autographiviruses and their relatives of other *Caudoviricetes* families.

## 1. Introduction

Bacteria of the *Aeromonas* genus are Gram-negative rods, widely distributed in a variety of environments, but they mainly inhabit aquatic habitats, such as rivers, lakes, ponds, seas, and wastewaters [1]. Some *Aeromonas* species have been found to be pathogens of warm-blooded animals, including humans [2]. They are also fish pathogens, causing septicemia and ulcerative diseases [3]. The most important *Aeromonas* species responsible for fish infection are *A. hydrophila* and *A. salmonicida* [4,5,6,7]. In view of the rapid emergence of antibiotic-resistant bacteria, new therapeutic approaches are being sought [8,9]. One of them is bacteriophage therapy [10,11].

Bacteriophages (phages for short) are viruses infecting bacteria, that are present in every environment. They are the most abundant biological entities in the biosphere, with estimated numbers of 10^31^ virions [12]. Due to their biological properties, phages have been considered potential antimicrobial agents [10,13]. Therefore, numerous studies about these natural predators of pathogenic *Aeromonas* spp. have been conducted to isolate and characterize phages that can be used in phage therapy [14,15,16,17]. Most of them were isolated from sewage and wastewater. Currently, 122 genomes of phages infecting *Aeromonas* strains are deposited in the NCBI GenBank database (20 February 2022). Twenty-four of them belong to the *Autographiviridae* family (Table S1). All members of the *Autographiviridae* family encode their own single subunit RNA polymerase and share a common unidirectional gene arrangement [18,19]. Based on the genome organization and RNA polymerase gene localization, four subgroups of the *Autographiviridae* family were distinguished—the T7-like, SP6-like, phiKMV-like, and P60-like phages [19]. For many years, representatives of *Autographiviridae* were considered strictly virulent, although a few putative prophage elements classified as T7-like *Autographiviridae* were identified in the *Xanthomonas axonopodis*, *Pseudomonas putida*, and *Burkholderia pseudomalle* genomes [20,21]. Recently, an autographivirus Pasto was found to be able to enter the lysogenic cycle in its host *Agrobacterium* sp. [22], making it the first active temperate *Autographiviridae* phage and starting point for the identification of T7-like prophages in a wide variety of Gram-negative and several Gram-positive bacterial genomes. Another functional temperate *Autographiviridae* was Teseptimavirus S2B, infecting the *Caulobacter crescentus* strain CB15 [23]. Nonetheless, all of the 24 known *Autographiviridae* phages infecting the genus *Aeromonas* are virulent (Table S1). The majority of them were isolated from polluted waters and sewage using the type strains (e.g., *Aeromonas salmonicida* subsp. salmonicida ATCC 33658, *Aeromonas hydrophila* ATCC 7966) and other fish or human pathogenic strains as hosts (Table S1). In this group, the only case when phages and their hosts were derived from similar sample isolation sites are Atoyac phages isolated from sewage and different rivers contaminated with sewage discharge in central, northern, and southern Mexico [24]. Therefore, there is not much information available about the natural host–parasite relationships between *Aeromonas* spp. and *Autographiviridae* phages.

Here, we report three novel virulent *Autographiviridae* phages, vB_AspA_Bolek, vB_AspA_Lolek, and vB_AspA_Tola, (henceforth referred to as Bolek, Lolek, and Tola, respectively) infecting different, yet closely related, *Aeromonas* spp. strains—MR7, MR19, and MR16, respectively. All of these host–pathogen pairs were derived from the same sampling place—microbial mats present in the arsenic-contaminated bottom sediments of the closed Zloty Stok gold mine (Western Sudetes, Poland), which was in operation from the early Middle Ages to 1961. Therefore, to our best knowledge, they are the first *Aeromonas*–autographivirus phage–host systems recapitulating natural relationships. Many indigenous bacteria of the Zloty Stok gold mine are not only arsenic-resistant but are also capable of environmental arsenic metabolism; for example, *Aeromonas salmonicida* strain O23A, the first representative of this genus able to perform dissimilatory arsenate reduction [25]. Following induction with mitomycin C, the phiO23A myovirus of the O23A strain was obtained [25]. Unlike temperate phiO23A, the three *Aeromonas* phages investigated in this study, Bolek, Lolek, and Tola, are virulent. They were characterized here in terms of their morphology, infection kinetics, and genomics, which turn out to be the starting point for the comparative analysis of genomes of the *Autographiviridae* family.

## 2. Materials and Methods

### 2.1. Bacterial Strains and Culture Conditions

Reasoner’s 2A (R2A) or lysogeny broth (LB) media and aerobic conditions were used for bacterial isolation and culture enrichment. Isolates were cultured in 50 mL of medium in flasks or on agar-containing plates at temperatures of 10–20 °C. The bacterial hosts of investigated phages, *Aeromonas* sp. MR7, MR16, and MR19 were isolated from microbial mats of the Zloty Stok gold and arsenic mine, collected in September 2018. The mat samples were diluted in 0.7% NaCl and directly plated on the R2A agar. The obtained colonies were used to isolate pure cultures. To obtain a number of the other bacteria from the microbial mat and biofilm samples of the Zloty Stok collected at different times (April or September 2018, February or July 2021), two methods were used. One was the same procedure as described above, and in the second, plating on the LB or R2A agar was preceded by 7 days of incubation of the mat or biofilm samples in LB or R2A media with shaking at 10 °C (Table S2). All other bacteria used in the host range spectrum determination (Table S2) were cultured on the LB or R2A agar at 20 °C.

### 2.2. Isolation of the vB_AspA_Bolek, vB_AspA_Lolek, and vB_AspA_Tola Phages

Phages vB_AspA_Bolek, vB_AspA_Lolek, and vB_AspA_Tola were isolated from microbial mats of the Zloty Stok mine collected at the same time, that is, September 2018, as were the samples used for the isolation of the *Aeromonas* sp. MR7, MR16, and MR19 strains using the method described previously [26]. For the long-term storage of the phage suspensions at 4 °C, saline magnesium buffer (100 mM NaCl, 50 mM of Tris HCl (pH 7.5), 8 mM of MgSO_4_ × 7H_2_O) was used.

### 2.3. Transmission Electron Microscopy (TEM)

TEM analysis was conducted as described previously [27]. The visualization of the phages was performed at the Core Facility of the International Institute of Molecular and Cell Biology (IIMCB, Warsaw, Poland).

### 2.4. Temperature Range of Bacterial Growth

The *Aeromonas* sp. MR7, MR16, or MR19 strains were plated with the use of the streak plate technique. The plates were incubated for 96 h at various temperatures ranging from 4 to 42 °C and examined after every 24 h. The data were obtained from three independent experiments.

### 2.5. Bacterial Growth Curve

LB or R2A media (9.8 mL) was mixed with 0.2 mL of the overnight cultures of the *Aeromonas* sp. MR7, MR16, or MR19. The cultures were grown at 10 or 20 °C with shaking (150 rpm). At appropriate time points, the OD_600_ absorbance was measured using a Sunrise Absorbance Reader with Magellan software (Tecan Austria GmbH, Grödig, Austria). The data were obtained from three independent experiments.

### 2.6. Testing the Influence of Various Temperatures on the Phage Plaque Formation

To test the influence of various conditions on the phage plaque formation, 10 µL drops of the Bolek, Lolek, and Tola phage suspensions, with a concentration of 10^11^ (10^8^ for Tola phage) to 10^2^ PFU/mL, were applicated onto the surface of double-layer R2A agar plates inoculated with the appropriate bacterial strain (*Aeromonas* sp. MR7, MR19, and MR16, respectively). In each case, the plates were incubated for 96 h at various temperatures from 4 to 37 °C and examined after every 24 h. The data were obtained from three independent experiments.

### 2.7. Thermal and pH Stability of the Phages

For thermal stability testing, 10 μL of the phage lysate (10^11^ PFU/mL for Bolek and Lolek, 10^8^ for Tola) was added to 990 μL of the R2A medium and incubated at 25, 30, and 40 °C. A control sample was incubated at 20 °C. After 10, 20, or 30 min, the numbers of PFU were determined by serial dilution of the phage mixtures and a plaque assay on R2A double agar plates with *Aeromonas* sp. MR7, MR19, and MR16 as hosts. Plates were incubated for 24 h at 20 °C. The data were obtained from three independent experiments.

For pH stability testing, 10 µL of the phage lysate (10^11^ PFU/mL for Bolek and Lolek, 10^8^ for Tola) was added to 990 µL of the R2A medium with different pH levels of 3, 5, 9, 11, and 13 (adjusted using NaOH or HCl), and incubated for 10, 20, or 30 min at RT. Control phage samples were incubated at pH 7 and RT, respectively. The numbers of PFU were determined by serial dilution of phage mixtures and plaque assay on R2A double agar plates with *Aeromonas* sp. MR7, MR19, and MR16 as hosts. The plates were incubated for 24 h at 20 °C. The data were obtained from three independent experiments.

### 2.8. Adsorption Kinetics

Adsorption assays were performed as described previously [28] but with modifications. The Bolek, Lolek, and Tola phage suspensions (10 µL; 10^8^ PFU/mL) were mixed with 990 µL of the overnight culture of the *Aeromonas* sp. MR7, MR19, and MR16, respectively, to obtain a multiplicity of infection (MOI) of 0.01. The phages mixed with hosts were incubated in LB and R2A media without shaking at 20 °C. After 2.5, 5, 10, and 15 min, samples were collected and centrifuged to remove the cells with adsorbed phage particles. The supernatant was used in a plaque assay on double agar plates with the same medium to determine the unabsorbed phage titer. In the case of the experiment with the Bolek and Lolek phages incubated with hosts in LB medium, R2A double agar plates were used for better visualization of the plaques. The plates were incubated for 24 h at 20 °C. The number of adsorbed phages was determined based on the ratio between the starting concentration of the phages added and the concentration of the unadsorbed phages. The data were obtained from three independent experiments.

### 2.9. One-Step Growth Curve

One-step growth curves were determined as previously described by Oliveira et al. [29] with modifications. LB or R2A media (9.8 mL) was mixed with 0.2 mL of the overnight cultures of the *Aeromonas* sp. MR7, MR19, or MR16. The cultures were grown at 20 °C with shaking (150 rpm) until OD_600_ = 0.2 and then infected with the Bolek, Lolek, or Tola phages at an MOI of 0.001. After 15 min of adsorption, the mixtures were centrifuged (5000× *g*, 1 min), suspended in 10 mL of fresh LB or R2A media, and further carried out at 20 °C with shaking (150 rpm). The number of phage-infected bacterial cells (infection centers, ICs) was determined at 1 min after infection as the numbers of PFU/mL by mixing 10 μL of the culture sample with an overnight culture of the host and 3 mL of top agar. Then, at the appropriate time points, the PFUs were determined by serial dilution of the suspension collected from the culture flask and standard plaque assay on double agar plates with the relevant medium. In the case of tests conducted in liquid LB medium, R2A double agar plates were used for better visualization of the Bolek and Lolek plaques. The plates were incubated for 24 h at 20 °C. The burst size was presented as PFU/IC. The data were obtained from three independent experiments.

### 2.10. Determination of Phage Host Range by Spot Testing

The phage host range was determined by an application of 10 µL drops of serial dilutions of the Bolek, Lolek, and Tola phage suspensions onto the surface of double-layer LB or R2A agar plates inoculated with the appropriate bacterial strain (Table S2) to obtained single plaques. The data were obtained from three independent experiments.

### 2.11. DNA Isolation and Sequencing

The total DNA of the *Aeromonas* sp. MR7, MR19, and MR16 strains were extracted from the overnight culture carried out in the LB medium with a Genomic Mini Kit (A&A Biotechnology, Gdansk, Poland). Whole-genome shotgun sequencing was conducted by Biobank Lab, University of Lodz (Lodz, Poland), on an Illumina MiSeq platform in the paired-end mode of 2 × 250 cycles.

The total DNA of the Bolek, Lolek, and Tola phages were isolated by phenol–chloroform extraction and isopropanol precipitation [30]. Phage genome sequencing was performed by Eurofins Genomics (Ebersberg, Germany) on an Illumina NovaSeq 6000 platform in the paired-end mode of 2 × 150 cycles.

The quality of each of the obtained datasets was investigated with FastQC v0.11.5 [31] and then processed with fastp v0.23.1 with the following parameters: cut_window_size 6; cut_tail; cut_mean_quality 19; length_required 50; n_base_limit 5; trim_poly_x; poly_x_min_len 10; correction; overlap_len_require 20; overlap_diff_limit 5; cut_front 15 [32]. The filtered reads were then assembled with SPAdes v.3.15.3 in a careful mode using the following k-mers list: 33,55,77,99,127 [33]. Assembled contigs were then subjected to the analysis of genome coverage based on the filtered reads, remapping with the application of bwa mem v.0.7.17-r1198-dirty [34] and samtools v.1.10 [35]. The genome coverage was then used to manually filter out bacterial contigs with low coverage. Moreover, the filtered reads were used for PhageTerm v.1.0.12 analysis to identify phage packaging systems [36].

### 2.12. Genome Annotation

The analysis of the nucleotide sequence of phages was performed using Clone Manager 8 (Sci-Ed) and Artemis v16.0.0 software [37]. The genomes were automatically annotated using the RASTtk [38] in phage mode on the PATRIC website [39]. The annotations were manually verified using the BLASTP (NCBI NR database) [40], HHpred (PDB_mmCIF70_12_Oct, SCOPe70_2.07, COG_KOG_v.1.0, Pfam-A_v35, and NCBI_Conserved_Domains (CD)_v3.18 databases) [41], Pfam [42], UniProt [43], and InterPro [44] databases. Putative transposable elements were identified using the ISFinder database [45]. Virulence factors were retrieved for each genome annotation from the specialty genes tab on the PATRIC website. Additionally, bacterial genomes were searched with the Prokaryotic Antiviral Defence LOCator (PADLOC) database and its webserver to discover putative defense systems [46].

### 2.13. Comparative Genomics Analysis

The comparison of bacterial strains and annotation of orthologous gene clusters among them were carried out using OrthoVenn2 using an e-value of 1 × 10^−15^ as a threshold [47]. Comparative genomics of the phage genomes was performed with the application of Clinker using the default settings [48]. If necessary, the genomes were circulated and reoriented to enhance the overview of genome structure conservation. The genomes of the Bolek, Lolek, and Tola phages were also compared with other known phage genomes recovered from the INPHARED database (as of the 1 December 2021 release) [49] with the application of vConTACT2 v0.9.20 as described previously [50]. All of the analyzed networks were visualized with Gephi v.0.9.2 [51], and the nodes were laid out in a two-dimensional space with the application of the ForceAtlas 2 [52] and Noverlap algorithms. For phylogenetic analysis of the major capsid and RNA polymerase proteins, these were first aligned with the default mafft v7.310 setting and further processed with IQ-TREE v1.6.1 using the LG+F+R7 model and 1000 replicates of UltraFast bootstrap analysis and the SH-aLTR test [53,54,55,56].

### 2.14. Nucleotide Sequence Accession Numbers

The draft genomes of the *Aeromonas* sp. MR7, MR19, and MR16 as well as the Bolek, Lolek, and Tola phage genomes can be accessed under the following GenBank accession Nos: JAKZFJ000000000, JAKZFL000000000, JAKZFK000000000, OM913597, OM913598, and OM913599.

## 3. Results and Discussion

### 3.1. Identification and characterization of the Hosts

The *Aeromonas* sp. MR7, MR16, and MR19 strains were isolated from microbial mats of the Zloty Stok gold and arsenic mine (see Section 2.1). The genomic DNAs of the MR7, MR16, and MR19 strains were isolated and sequenced. The reconstruction of their genomes resulted in 54–97 contigs with a total length of 4,655,773–4,883,405 (Table 1). The overall GC content of the chromosomes is 60.2–60.9%, which is consistent with the other sequenced *Aeromonas* genomes, with GC content ranges between 58.17% and 62% [57]. The analysis of the MR7, MR16, and MR19 genomes with the RAST annotation system available on the PATRIC platform [38,39] revealed the presence of antibiotic resistance genes and putative virulence factors that indicate their virulence potential despite being environmental isolates (Table 1 and Table S3). Three prophages were identified in the MR16 genome and one in the genomes of MR7 and MR19 (Table 1). Additionally, a putative extrachromosomal element (3241 bp), similar to the pO23AP1 plasmid of the *Aeromonas salmonicida* strain O23A (GenBank CP021655.1), was found in the MR7 genome. Apart from prophages and plasmids, the mobilomes of MR7, MR16, and MR19 comprise a number of putative transposable elements. In total, 14–52 genes encoding transposases were found (Table 1). According to the ISFinder database [58], they belong to nine families—IS110, IS1595, IS256, IS3, IS4, IS481, IS5, IS66, IS91, and ISNCY. Insertion sequences belonging to similar families were identified in the O23A genome, also originating from the Zloty Stok mine. The *Aeromonas salmonicida* strain O23A comprises 22 genes encoding the transposases of transposable elements belonging to eight families [59], among which five are the same as these identified in the MR7, MR16, and MR19 strains. The overall comparison of the MR7, MR16, and MR19 genomes with O23A revealed 3214 of 3559–4142 common protein clusters (Figure S1).

Since our goal was to use the MR7, MR16, and MR19 strains as hosts for viruses, we performed the mining of their genomes for defense potential. The annotation of the genomes and the Restriction Enzyme Database (REBASE) [60] allowed us to identify one putative Type I restriction–modification (RM) system in each genome, showing, in the case of MR7 and MR16, similarities to known RM systems that differ with respect to the target sequences AspNIH5II (CAAGN_7_CTGC) and Aca75I (ACAGN_5_CTA), respectively. In the MR19 genome, we found not only a Type I, but also a Type II RM system with putative CTGCAG sequence specificity. Further, we used the Prokaryotic Antiviral Defense LOCator (PADLOC) database and its web server [46] to discover many more putative defense systems, for example, in MR7, a bacteriophage exclusion defense system (BREX) that blocks phage replication and discriminates between host and phage DNA by methylation patterns [61,62]; in MR19, two retron systems that employ various specialized reverse transcriptases fused at the C-termini with different domains (TIR or TOPRIM) [63,64]; in MR16, a membrane-associated kiwa system [65] and pyrimidine cyclase system (Pycsar) [66]. It is worth emphasizing that each analyzed genome hosted a variety of defense systems for protection against foreign DNA, and neither was present in any other strain (Table S4). We have not found any CRISPR system within the MR7, MR16, and MR19 genomes, which in turn was detected in O23A [25]. Although some of its CRISPR spacers matched several *Aeromonas* phages, none of them showed sequence similarity to the genome sequences of phages identified in this study.

Analyses performed on the plates with LB and R2A media showed that MR7, MR16, and MR19 could grow at various temperatures ranging from 4 to 37 °C (Table S5), similar to the O23A strain; however, unlike it, they were not able to grow at 42 °C [59]. Moreover, the growth curves of MR7, MR16, and MR19 strains show that the LB liquid cultures reached OD_600_ = 0.3 after 7 h of incubation at 20 °C and after 18–21 h incubation at 10 °C (Figure S2). Nonetheless, there was no significant difference between the generation times in the LB versus the R2A media at 20 °C (about 3 h). At 10 °C, the LB liquid medium cultures of all three strains had generation times of about 3–4 h; however, in the R2A, cell doubling was achieved only by MR19 (after 5 h). In summary, despite the ability of the studied strains to grow at low temperatures (they were isolated from a constant low-temperature environment and showed this capacity in the laboratory condition), the MR7, MR16, and MR19 strains had optimal growth temperatures of ~20°, and therefore, can be considered psychrotolerant [67]. In addition, they seemed to prefer nutrient-rich media, as they grew faster in LB versus R2A, although they must also be well adapted to the nutrient-sparse environment from which they originated.

### 3.2. Identification and Functional characterization of Phages

#### 3.2.1. Identification of the vB_AspA_Bolek, vB_AspA_Lolek, and vB_AspA_Tola Phages

**Figure 1:**
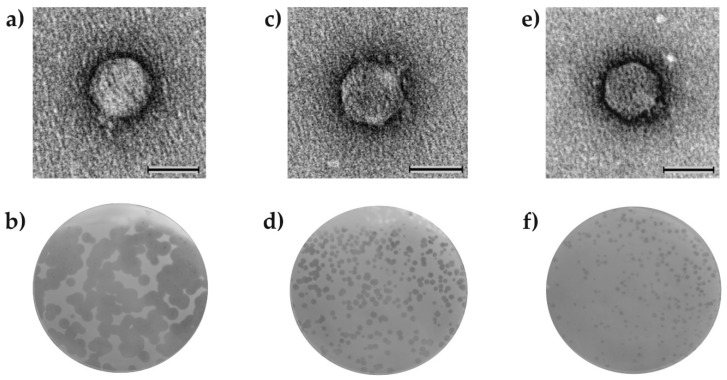
Visualization of the virion and plaques of the (**a**,**b**) Bolek, (**c**,**d**) Lolek, and (**e**,**f**) Tola phages. The scale bar represents 50 nm (**a**,**c**,**d**).

The *Aeromonas* sp. MR7, MR19, and MR16 strains were used as potential hosts for bacteriophages from the microbial mat samples of the Zloty Stok gold mine, which resulted in the isolation of the vB_AspA_Bolek, vB_AspA_Lolek, and vB_AspA_Tola phages called Bolek, Lolek, and Tola for short. Electron micrographs of the negatively stained virions (Figure 1a,c,e) showed icosahedral heads of approximately 66–68 nm, with very short tails. This *Podoviridae*-like tail-morphotype together with genomic analysis (i.e., the presence of genes encoding RNA polymerase; see Section 3.3), indicated that Bolek, Lolek, and Tola could be classified in the *Autographiviridae* family.

The Bolek, Lolek, and Tola formed clear plaques (characteristic of virulent phages) on host lawns grown on R2A agar plates, but when the LB agar plates were used instead, the Bolek and Lolek plaques were poorly visible, whereas the Tola phage formed clear, good visible plaques regardless of the medium used for its host MR16 cultivation (Figure S3). The sizes of the Bolek, Lolek, and Tola plaques were measured on the appropriate host lawn grown on R2A agar plates incubated for 72 h at 20 °C. The biggest plaques were formed by the Lolek phage (3–5 mm in diameter), while the smallest plaques (1–2 mm) were formed by the Bolek (Figure 1b,d,f and Figure S4). The Bolek plaques were surrounded by an opaque-looking halo zone that appeared 24 h after the plaque formation and increased upon prolonged incubation (Figure 1b and Figure S4). The halo zone indicated the presence of depolymerase—a virion-associated enzyme responsible for the degradation of the bacterial exopolysaccharide layer [68]. The role of the depolymerase is probably performed by the Bolek_p50 protein in which the pectate lyase domain was identified (see Section 3.3).

#### 3.2.2. The Influence of Various Temperatures on the Phage Plaque Formation

To check how various temperatures affected the plaque formation of the Bolek, Lolek, and Tola phages, we used the double agar overlay technique. The Bolek, Lolek, and Tola phages generated plaques on the R2A bacterial lawn when their hosts were cultivated at 4–30 °C; however, no plaques were observed at 37 °C, while their hosts were able to grow at this temperature (Table S5) and formed lawns. The ability to multiply at low temperatures (4–10 °C) allows us to consider Bolek, Lolek, and Tola as cold-active phages. The time of the plaque appearance of all three phages was related to the time of bacterial lawn formation, which in turn was associated with the incubation temperature used; at 20–30 °C, the plaques were observed after 24 h, at 10 °C after 48 h, and at 4 °C after 96 h. In the case of the Bolek phage, growing turbid halos around plaques appeared approximately 24 h after the plaque formation at 10 °C and 20 °C. However, no halo was seen at 4 °C and 30 °C even after 96 h of incubation. The simplest explanation for this result could be the narrow temperature range over which the enzyme remains active. It was shown that several depolymerases of *Klebsiella* autographiviruses were stable and fully active under a wide range of temperatures, unlike KP36 (a representative of the Webervirus genus), which was not temperature resistant [68,69]. On the other hand, the plaques of ZPAH7, N21, and G65 infecting *A. hydrophila* [70,71,72] and vB_AsM_ZHF of *A. salmonicida* [73] were surrounded by a turbid zone, but no experimental studies of depolymerases encoded by these phages are available. It is suggested that halo formation is caused not only by the excess of exopolysaccharide depolymerases produced inside the host during phage replication and released upon bacterial lysis but also by viral diffusion out of the lysis zone of primarily infected bacteria; thus, the presence and extent of halo would also depend on the number of phages produced in one single plaque [74]. It is, therefore, possible that the number of the Bolek phage particles produced at 4 °C and 30 °C is very low compared to the production at 10 °C and 20 °C.

#### 3.2.3. Sensitivity to Temperature and pH of the Bolek, Lolek, and Tola Phages

**Figure 2:**
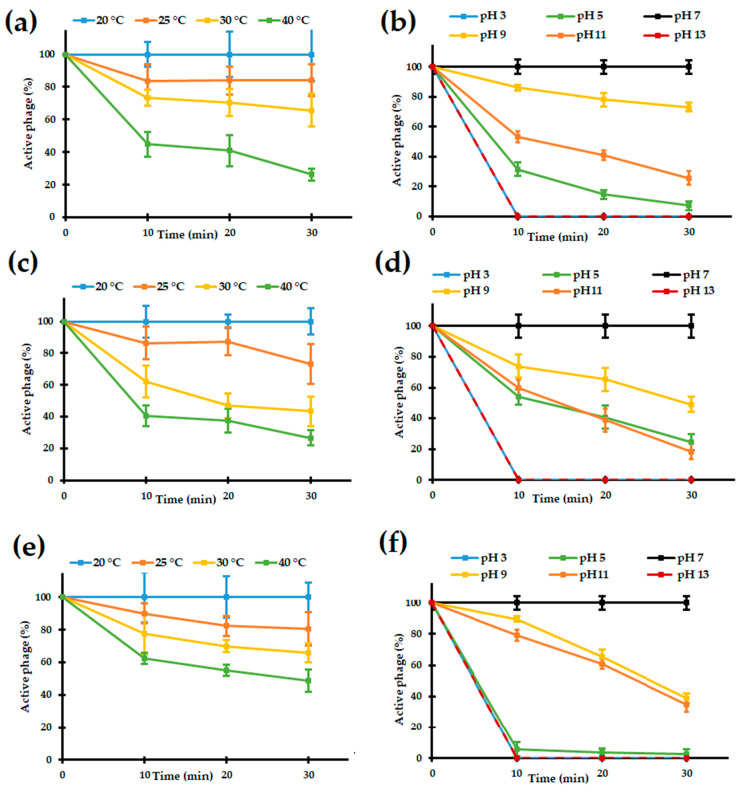
(**a**,**b**) Bolek, (**c**,**d**) Lolek, and (**e**,**f**) Tola phage stability under various temperatures and pHs. Temperatures and pHs are indicated according to the legends.

To characterize the influence of physico-chemical factors on the Bolek, Lolek, and Tola, the thermal and pH stability of these phages were checked. Thermal stability tests showed that the Bolek, Lolek, and Tola phages were sensitive to temperatures above 25 °C (Figure 2a,c,e). After 30 min of incubation at 30 °C, only 45–70% of the phage particles remained active, while incubation at 40 °C resulted in an even faster loss of phage infectivity. Moreover, the Bolek, Lolek, and Tola were sensitive to pH levels lower and higher than 7 (Figure 2b,d,f). Incubation of the Bolek and Lolek phages at pH 3 and 13 resulted in the immediate loss of activity, as did the incubation of the Tola phages at pH ≤ 5 and 13, which makes the Tola phages more sensitive to acidic conditions. Based on these results, we can conclude that Bolek, Lolek, and Tola hardly tolerate conditions different from those prevailing in the Zloty Stok mine, that is, a temperature ~10 °C and slightly alkaline water (pH 7–8) [75,76]. Similarly, limited tolerances to these physicochemical factors were previously shown for *Serratia* BZS1 and *Shewanella* KASIA phages that were also isolated from the Zloty Stok mine [26,27].

#### 3.2.4. Adsorption and One-Step Growth of the Bolek, Lolek, and Tola Phages

In order to verify whether the media richness influenced the effectiveness of the adsorption and propagation of the Bolek, Lolek, and Tola phages, we performed adsorption and one-step growth assays in both LB and R2A media. Due to the poor visibility of the Bolek and Lolek plaques formed on their host lawns cultivated on LB medium (see Section 3.2.1), plaque assays used to estimate the adsorption kinetics and one-step growth of the Bolek and Lolek phages were carried out on R2A double agar plates.

**Figure 3:**
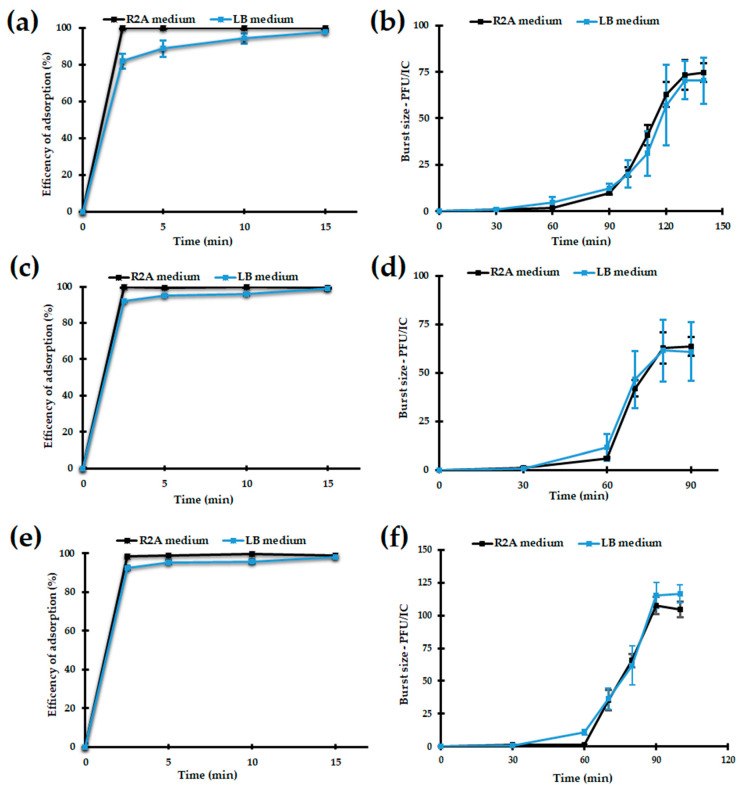
(**a**,**b**) Bolek, (**c**,**d**) Lolek, and (**e**,**f**) Tola phage adsorption to the bacterial cells and one-step growth curves in R2A and LB media.

Adsorption experiments showed that in both media, the studied phages adsorbed quickly to host cells—after 2.5 min, >90% of the Bolek, Lolek, and Tola phage particles were attached to the MR7, MR19, or MR16 cells, respectively (Figure 3a,c,e). On the other hand, adsorption tests were carried out in the same conditions as for the host strains on which the studied phages were isolated showed that Bolek, Lolek, and Tola were able to adsorb exclusively to the MR7, MR19, and MR16 cells, respectively (Table S6). These results suggest that adsorption might be a crucial step in dictating the studied phages’ host range specificity.

One-step growth assay revealed almost no differences between the propagation of the Bolek, Lolek, and Tola phages in their appropriate host cells cultivated in LB and R2A media. The latency periods, rise periods, and burst sizes of these phages were almost the same regardless of the medium used (Table 2). Although it might be expected that the metabolism of the host cultivated in rich versus nutritionally reduced media would result in differences in the rate of reproduction of a virus or its titer, it did not significantly affect the efficiency of the Bolek, Lolek, and Tola multiplication. However, it is worth noting that the Bolek, Lolek and Tola phages were heterogeneous in rise periods (20–70 min) and burst sizes (~60–114 PFU/IC) (Table 2) which can be explained by multiplication in different hosts.

#### 3.2.5. Host Range

Numerous studies have demonstrated that bacteriophages, particularly members of the *Autographiviridae* family, have a narrow host range [77,78,79,80]. On the other hand, some of the phages, including representatives of *Autographiviridae*, are able to infect multiple strains of the same species of bacteria (e.g., *Aeromonas* PZL-Ah8) or infect multiple species within the same genus (e.g., *Aeromonas* PZL-Ah1) or polyvalent ones able to multiply on different bacterial genera [81]. An exceptional example of polyvalent viruses within *Autographiviridae* is a group of closely related environmental Atoyac phages infecting bacteria from six genera—*Aeromonas*, *Pseudomonas*, *Yersinia*, *Hafnia*, *Escherichia*, and *Serratia* isolated from the similar sources where the phages originated [24] (Table S1). Given the possibility that the studied phages also have a promiscuous host range, we used a taxonomically diverse panel of 71 bacteria native to the Zloty Stok gold mine as potential hosts and spot tested with undiluted phage stocks. The results show that only those strains used for the initial plaque formation by Bolek, Lolek, and Tola, and a few other *Aeromonas* spp. isolates from the mats turned out to be susceptible to infection by the tested phages (eight for Bolek, five for Lolek, two for Tola). However, it should be emphasized that cross-infection assays failed; that is, none of the *Aeromonas* strains sensitive to Bolek were susceptible to infection by Lolek and Tola, none sensitive to Lolek were susceptible to Bolek and Tola, and none sensitive to Tola were susceptible to Bolek and Lolek. Interestingly, the hosts sensitive to Bolek, Lolek, or Tola were found not only on the same but also on different sampling dates (Table S2). No plaque formation was observed for either the phage on overlays of the 45 other *Aeromonas* strains (including O23A) or the bacteria of other *Proteobacteria* genera originated from Zloty Stok mats or biofilm. Moreover, none of the *Aeromonas*-type strains from the German Collection of Microorganisms and Cell Cultures (DSMZ) used in this study supported detectable lytic growth of either of the tested phages. In summary, the studied phages, Bolek, Lolek, and Tola, seemed to be highly host-specific, which is, as mentioned above, quite a distinctive feature of the *Autographiviridae* members known so far. Although it cannot be excluded that the MR7, MR16, and MR19 strains are hosts for other viruses of Zloty Stok than those tested or that the Bolek, Lolek, and Tola phages infect a significantly larger number of strains (e.g., those that are not cultivable in a laboratory), the very intriguing question remains why viruses occupying the same niche do not use all three strains for multiplication, which, as shown in Section 3.1, seem to be quite similar to each other (Figure S1). Having a broad range of hosts by a virus is a competitive advantage in oligotrophic systems where host diversity may be high, but abundance is low [82].

### 3.3. Genomic characterization of Phages

The genomes of all three studied phages are double-stranded DNA, with similar sizes of 42,433, 43,136, and 45,276 bp for the Bolek, Lolek, and Tola, respectively. Based on their genome sequence comparison and virion morphology, these phages were classified as representatives of *Autographiviridae*. The GC content of the Bolek, Lolek, and Tola genomes was from 47.4% (Tola) to 52.7% (Bolek), which is around 10 percentage points less than the average of their host genomes, respectively (Table 1; Table 3). The Bolek, Lolek, and Tola genomes were predicted to contain 57–70 putative genes (Table 3), which were located on the sense strands. The function was assigned to 27–32 of their predicted products (44–46% of the total number of genes), while the remaining ones were classified as hypothetical proteins (Table 3). No gene encoding tRNA was detected in their genomes. The annotations of the Bolek, Lolek, and Tola genomes are provided in Tables S7–S9, respectively. The analysis of the genome sequence coverage and application of the PhageTerm tool [36] revealed the presence of 314, 505, and 631 bp-long non-permuted direct terminal repeats at the end of the Bolek, Lolek, and Tola genomes (Files S1–S3), yielding their physical genomes with sizes of 42,747 bp, 43,136 bp, and 45,907 bp, respectively.

Based on the analysis of 944 *Autographiviridae* phage genomes, we conclude that almost all known *Autographiviridae* phages have genomes in the size range of 35–50 kb, making the Bolek, Lolek, and Tola phage genome lengths typical for known *Autographiviridae* viruses (Figure S5). However, the Tola phage has the largest genome of the known *Autographiviridae* members infecting *Aeromonas* spp. (Table S1). Similar to other *Autographiviridae*, the Bolek, Lolek, and Tola phages all have genes encoded solely on the Watson strand and short exact repeat ends. In addition to the quite narrow genome size range and the terminal repeats, another conserved feature of *Autographiviridae* is gene synteny, and the presence of the RNA polymerase gene (RNAP) that is considered to be the hallmark of this family [19]. Comparing the Bolek, Lolek, and Tola with other genomes of *Autographiviridae* phages and considering putative gene functions, three functional genomic regions can be distinguished—the early (host conversion) region, the DNA replication and metabolism region, and the morphogenesis region. RNAP genes encoded by Bolek and Lolek (*Bolek_p37, Lolek_p38*) are located downstream of the DNA replication and metabolism region (the same as in phiKMV-like phages), while Tola’s RNAP gene (*Tola_p19*) is located between the early and replication module typical for SP6-like phages.

The early regions of all three phages comprise numerous small genes that encode hypothetical proteins that might be involved in host shutoff. The early region of the Bolek and Lolek phages encode 19 and 18 proteins with unassigned functions (Bolek_p01-_p20, Lolek_p01-_p19), among which 13 proteins have no similarity to other gene products in the NCBI database. We were able to assign functions only to the putative early proteins Bolek_p15 and Lolek_p14, which might act as tRNA-nucleotidyltransferases (TRNT), which function with an unusual mechanism of polymerization, adding the nucleotide triplet CCA to the 3′-end of tRNAs [83]. Genes encoding TRNT have been found in many bacteriophages, including *Autographiviridae* viruses. Evseev et al. [84] found out that the TRNT gene was present in most genomes of *Pectobacterium* phages belonging to different *Autographiviridae* subfamilies (80% of those known at that time) and suggested considering it a possible hallmark of Pectobacterial *Autographiviridae*. On the contrary, the TRNT genes seem to be a rather rare feature of *Aeromonas* autographiviruses, as only 33% (including Bolek and Lolek phages) of them possess a TRNT gene. A BLASTP examination of the Bolek_p15 and Lolek_p14 showed very low-sequence similarity (<30%) to TRNTs of *Autographiviridae* phages infecting *Pseudomonas*. No TRNT encoding gene was found in the Tola genome. Apart from the RNAP gene *(Tola_p19),* the early region of Tola contains 25 genes encoding hypothetical proteins (*Tola_p01-_p26*), of which 18 showed no significant similarity to the others in the NCBI database. No gene encoding integrase or CI repressor, characteristic of early gene regions of temperate phages, were detected in the Bolek, Lolek, and Tola genomes.

The analysis of the DNA metabolism regions of the Bolek, Lolek, and Tola phages revealed the presence of several genes encoding proteins presumably involved in replication—DNA primase, DNA helicase, DNA ligase, DNA polymerase, DNA 5′-3′exonuclease, and DNA endonuclease VII. Interestingly, the localization of putative DNA ligase genes is different in the Bolek, Lolek, and Tola. The Bolek and Lolek ligase genes (*Bolek_p27*, *Lolek_p26*) are located between the DNA helicase and DNA polymerase genes, which is characteristic, again, for the phiKMV-like group of phages, while the ligase gene of Tola (*Tola_p44*) is located immediately upstream the structural genes, which is a distinguishing feature of the SP6 subgroup. Moreover, in these regions, DUF669-containing proteins (predicted as distant homologs of single-strand DNA-annealing proteins involved in DNA replication [85]), phosphatase 2a-like proteins, polynucleotide 5′ kinases, and ATPases were identified.

The late gene regions of the Bolek, Lolek, and Tola phages contain at least 10–13 genes encoding putative structural proteins. The most interesting among them seem to be tail fiber proteins, as they play a key role in adhesion receptor-mediated virion attachment and entry into the host cell. The Bolek_p50, Lolek_p54, Tola_p58, and Tola_p69 proteins were annotated as tail fiber proteins. The first three shared the T7-like N-terminal domain (pfam03906) with the gp17 of *Escherichia* T7 phage (Figure S6). For T7, it was demonstrated that this part of the gp17 connected with the baseplate [86]. The mutual similarities between the N-terminal segments (~148 aa) of both Bolek_p50 and Lolek_p54 and Tola_p58 were 67% and 31%, respectively (Figure S6). An HHpred search for Bolek_p50 identified a hit to pectate lyase superfamily proteins (PF12708). We suppose that this protein is responsible for the generation of halo zones around the Bolek plaques (Figure 1b and Figure S4), although this hypothesis needs experimental validation. It is also possible that Lolek and Tola have their own depolymerases not expressed under laboratory conditions (no halos around the Lolek and Tola plaques were observed at any temperature tested; Figure 1d,f) since the InterPro tool identified the beta helix domain, characteristic of depolymerases [86] in Lolek_p54 (Figure S6). Meanwhile, the pectate lyase domain, predicted to be responsible for depolymerase activity, was identified in the second tail fiber protein of Tola—Tola_p69. Therefore, the Tola phage fiber seems to consist of two different proteins, similar to the *Salmonella* bacteriophage SP6. It was suggested that the two tail-fiber-like proteins of the SP6 phage (gp37 and gp46) function as a complex of both types of subunits, with gp37 forming a linkage between the virion and the receptor-binding gp46 protein [87]. Thus, we suppose that the Tola_p58 and Tola_p69 are counterparts of the gp37 and gp46 of SP6. Additionally, these proteins have the same localization in the Tola and SP6 genomes and share a similar gene context (Figure S7).

In the late gene regions of Bolek, Lolek, and Tola, we also distinguished two genes encoding putative terminase subunits and the lysis cluster. Bolek_p55 and Lolek_p58 were classified as putative endolysins, as they possess the peptidase M15 domain (pfam13539) also present in the L-alanyl-D-glutamate endopeptidase of *Escherichia* phage T5 [88], whereas Tola_p65 probably plays a similar role because it showed similarity with its counterpart of *Pectobacterium* phage PP99 [89] and *Pseudomonas* phage MR4 [90]. All other products encoded by genes in putative lysis clusters contain transmembrane helices (Bolek_p56, Bolek_p57, Lolek_p59, Lolek_p60, Tola_p59, Tola_p62, Tola_p67, and Tola_p68). These may act as holins and spanins.

### 3.4. Comparative Analysis of the Bolek, Lolek, and Tola Phages

The mutual comparison of the nucleotide sequences of the Bolek, Lolek, and Tola genomes showed that only Bolek and Lolek are similar to each other at a significant level, that is, with a query coverage of 84% and sequence identity of 92.72%. The most variable segments of the Bolek and Lolek genomes are in genes encoding hypothetical proteins mostly located in the early regions and tail fiber genes (Figure S8). Similar observations were made for other *Autographiviridae* representatives, that is, Fri-like phages infecting *Acinetobacter* (AS11, AS12, Fri1, vB_AbaM_IME200, Abp1, phiAB1, phiAB6, vB_AbaP_PD-AB9, and vB_AbaP_PD-6A3) were highly similar at the DNA level but differed in early genes and genes encoding tail fiber proteins [91].

**Figure 4:**
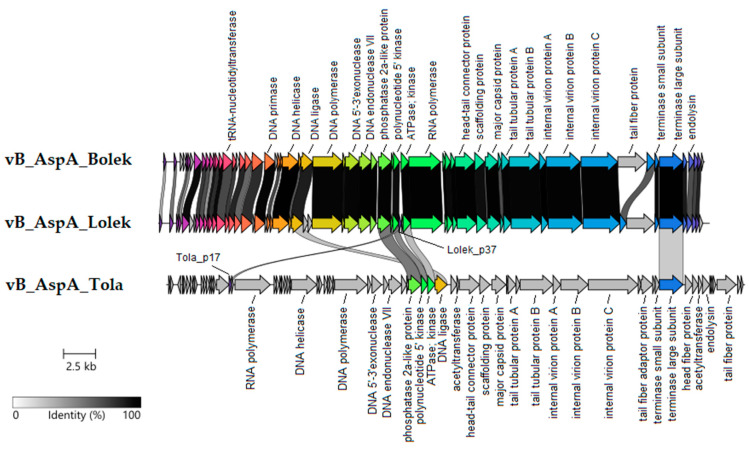
Comparative genome alignment of the Bolek, Lolek, and Tola phages. Coding sequences are represented by arrows, colored to reflect homologous ones, and are linked by grey bars shaded to represent the percentage of amino acid identity, as indicated in the legend.

Protein-based sequence similarity analysis conducted with Clinker [48] showed that the Bolek, Lolek, and Tola phages share a sequence similarity (of at least 30% sequence identity) among four proteins involved in DNA metabolism (phosphatase 2a-like protein, polynucleotide 5′ kinase, ATPase, DNA ligase) and terminase large subunits (Figure 4). Moreover, Lolek and Tola share one small hypothetical protein (Lolek_p37 and Tola_p17) whose counterpart is not present in the Bolek genome (Figure 4) [92]. Although the Bolek and Lolek phages encode several unique small hypothetical proteins (Figure 4; CDSs without labels), they share 51 of 57–61 proteins (83–89%) (Figure 4). In addition, the Bolek and Lolek tail fiber proteins (Bolek_p50 and Lolek_p54) (except for their N-terminus—see Section 3.3) demonstrate a significant difference in the amino acid sequence. It was shown before that the host range is often associated with specific interactions between the C-terminal end of the tail fiber protein and the receptor on the susceptible host cell [93,94]. In particular, host range modification of T7-like phages has been achieved through the exchange of C-terminal domains within their tail fiber genes [95]. Thus, the differences in the amino acid sequences of the C-terminal part of the tail fiber proteins of Bolek and Lolek and the nonhomologous tail fiber protein Tola_p69 (see Section 3.3, Figure S6) may explain the different host preferences of the studied phages. In particular, this might be an explanation for the adsorption test results (see Section 3.2.4), which showed that Bolek, Lolek, and Tola were able to adsorb exclusively to the MR7, MR19, and MR16 cells, respectively (Table S6).

### 3.5. Comparative Analysis of the Bolek, Lolek, and Tola with Other Phages

A comparison of Bolek, Lolek, and Tola at the nucleotide level with the content of the NCBI nucleotide database using BLASTN revealed that they all share low nucleotide sequence coverage and identity with other known phage genome sequences showing their distinctness. In particular, the genome coverage ranged between 0.7 and 10%, and sequence identity ranged between 71 and 75%. Since each of the studied phages showed a very low degree of nucleotide sequence similarity to known viruses, and even Bolek and Lolek differ more than 5% from each other at the nucleotide level. These three phages can be considered as three novel species within *Autographiviridae* based on the current ICTV guidelines (criterion of 95% DNA sequence identity for the demarcation of species) [96,97].

**Figure 5:**
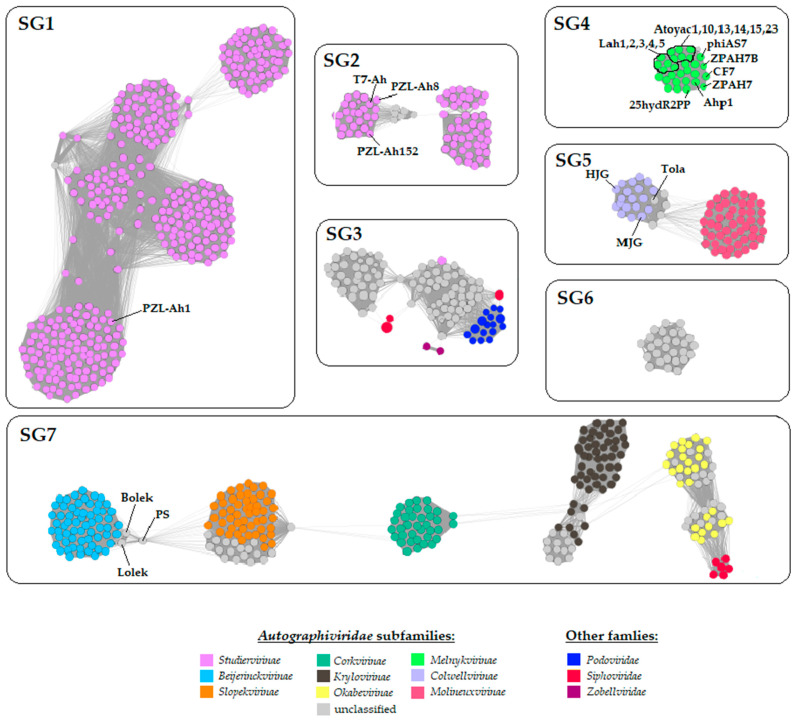
Protein-based phage similarity network. Each circle (node) represents a genome and connecting lines (edges) represent the similarity between genomes based on shared clusters of proteins. Only *Autographiviridae* family members and phages of other families demonstrating significant similarity to them are shown. Bolek, Lolek, Tola, and other *Aeromonas* phages are indicated with black lines and names. The coloring of the nodes (according to the key) is based on the current taxonomic classification of the phage, that is, subfamilies of *Autographiviridae* members and families of other phages. The edge thickness reflects the degree of similarity between two phages (the thicker the edge, the higher the similarity), considered as an overall similarity of proteins encoded by a pair of phages.

In order to further examine the relationship between Bolek, Lolek, and Tola and to refine their taxonomic status, a protein-based phage network was generated with vConTACT2 [50] and the INPHARED database (1 December 2021 release, over 17,470 phage genomes) [49]. From the resulting network, all phages classified as *Autographiviridae* (or the ones connected with them via edges) were retained, which resulted in a final network of 972 phages, among which 944 belonged to the *Autographiviridae* family (including the Bolek, Lolek, and Tola ones) (Figure 5). Most of these phages (919 of 944) infect Gram-negative bacteria, that is, *Alphaproteobacteria* (79), *Betaproteobacteria* (34), *Gammaproteobacteria* (804, including 27 *Aeromonas* hosts), and *Cyanophyceae* (31) (Figure S9). Two autographiviruses infect Gram-positive bacteria of the *Bacilli* class (*Enterococcus* phages EFA-1,MT350292.1 and EFA-2,MT350293.1), and the host of the 23 others is unknown (their genomes were derived from metagenomic studies). The presented analysis comprised a significantly higher number of *Autographiviridae* genomes than any previous comparative genomic studies of this family; therefore, we decided to take advantage of this opportunity and evaluate these data in addition to determining the taxonomic affiliation ofBolek, Lolek, and Tola.

Within the protein-based phage similarity network, seven independent subgraphs (Figure 5; SG1–SG7) were distinguished, and within them, multiple cliques (groups of closely related phages) were observed. The majority of phages located in SG1 and SG2 (composed of four and three cliques, respectively) and one phage from SG3 were classified as the most numerous subfamily of *Autographiviridae*–*Studiervirinae*. Among the representatives of the *Studiervirinae* subfamily, there were four *Aeromonas* phages—one within SG1 and three within SG2. Within these three subgraphs (and other subgraphs in Figure 5), several phages unassigned at the subfamily level could also be found, and we propose here to classify some of them into particular taxonomic ranges (mostly to subfamilies) according to their already classified neighbors. For example, the localization within the network and vConTACT status suggest that *Pasteurella* phage vB_PmuP_PHB02 of SG1, *Delftia* phage IME-DE1, and five *Ralstonia* phages located in SG2 could be assigned to the *Studiervirinae* subfamily (Figure 5; colored grey; Figure S10, Table S10). In the case of SG3, which consisted mostly of unclassified viruses, there was one recently described *Studiervirinae* phage [23], Teseptimavirus S2B, which implies that other phages of at least the S2B clique might be its subfamily relatives. However, it must be noted that unlike other subgraphs comprising *Studiervirinae* phages, SG3 was more heterogeneous as it consisted of not only unclassified autographiviruses (of which many were obtained from metagenomic studies) but also *Sipho*-, *Podo*-, and *Zobellviridae* phages. Two *Roseobacter* zobellviruses, CRP-4 and CRP-5 [98] are similar to the *Pelagibacter* autographivirus HTVC031P [99] through an endonuclease and two hypothetical proteins (Figure S10). Additionally, HTVC031P shares an exonuclease and DNA polymerase with CRP-5 and CRP-4, respectively. (Figure S11). Thirteen *Pelagibacter* podo- and 22 autographiviruses, together with seven uncultured phages, shared a few small proteins with unknown functions (Figure S12). In addition, the same autographiviruses shared a DNA polymerase and primase with the other three *Pelagibacter* podoviruses (Figure S12). Furthermore, the *Pelagibacter* siphovirus Kolga [100] and more than 20 autographiviruses showed significant sequence identity over a DNA primase, ribonucleotide diphosphate reductase subunits, and a few small hypothetical proteins (Figure S13). Moreover, two *Synechococcus* siphoviruses, KBS-S-2A [101] and S-CBS4 [102], were similar to *Synechococcus* autographiviruses S-SRP02 [103], S-B28 (NC_048171.1), and S-SRP01 [104] through a few hypothetical proteins (Figure S14). It has not escaped our attention that the above-mentioned phages of the SG3 subgraph infect similar hosts, that is, marine bacteria of the *Alphaproteobacteria* class (often of the same taxonomic genus). Therefore, in the future, it would be interesting to verify whether these shared proteins (especially those with unknown functions) allow the phages to “take over” host functions.

Another subgraph, SG4, creates a dense clique of phages assigned to the *Melnykvirinae* subfamily, among which the majority of known *Autographiviridae* phages infecting *Aeromonas* (17) were located. It is worth noting that these *Aeromonas* autographiviruses were isolated from seven geographically distant places (Australia, India, Mexico, China, Poland, Taiwan, and South Korea, Table S1).

Within SG5, there are two cliques of *Colwellvirinae* and *Molineuxvirinae* representatives exclusively connected to each other through four *Gajwadongvirus* members (unassigned so far at the subfamily level) acting as bridges. Here, Tola clustered with the 19 *Colwellvirinae*, among which there were two *Aeromonas* phages (MJG [105] and HJG, MK455770.1) and the four above-mentioned *Gajwadongvirus* phages (Figure 5; SG5; Table S11). The Clinker alignment showed that Tola shared 30–33 and 20 out of 52 proteins with *Gajwandongviruses* and *Colwellvirinae* members, respectively (Figure S15). In both cases, these were proteins involved in DNA replication and metabolism and proteins encoded by genes located in the structural module; however, polynucleotide 5′ kinase and ATPase, internal virion proteins B and C, the tail fiber adaptor, and the head fiber were exclusively similar to Gajwadongviruses’ proteins (Figure S15). The vConTACT analysis classified both Tola and *Gajwadongviruses* with the same overlap status, and the insightful inspection of their genome alignments revealed high similarity between the proteins they encode. This allows us to consider Tola as another representative of the *Gajwadongvirus* genus. Furthermore, considering the fact that the number of *Gajwadongviruses* increased from two to five in comparison with the last ICTV taxonomic ratifications [96], the degree of protein similarity combined with the analysis of genome alignments of *Gajwadongvirus* with *Colwellvirinae* and *Molineuxvirinae* phages (between which they act as bridges) suggests that *Gajwadongvirus* should be considered another genus of the *Colwellvirinae* subfamily (Table S10). This claim of assignment of Tola and other gajwadongviruses to the *Colwellvirinae* subfamily is strongly supported by the results of the phylogenetic trees constructed using RNA polymerase and major capsid proteins (Files S4 and S5).

The SG6 comprises only phages that have not yet been assigned to any subfamily. Among these, there are 23 phages infecting *Alphaproteobacteria* (*Rhizobium* spp., *Agrobacterium* spp., *Stappia* spp.) and 5 infecting *Gammaproteobacteria* (*Vibrio* spp., *Alteromonas* spp.) (Figure 5; colored grey; Table S12). Since SG6 clearly distinguishes itself from the remaining subgraphs (subfamilies) and gathers 28 presumably related *Autographiviridae* phages, it could be considered a candidate for a novel subfamily.

Finally, SG7 is composed of viruses belonging to five *Autographiviridae* subfamilies (Figure 5) organized in separate cliques connected with others through single viruses acting as bridges and emphasizing the mosaic feature of these phage genomes. The Bolek and Lolek phages are located in SG7. Bolek and Lolek gather with 54 *Beijerinickvirinae* representatives (most of which are almost identical *Acinetobacter* phages) and with the *Aeromonas* phage PS (uncharacterized and yet unclassified autographivirus, MT259468.1), which acts as a bridge between the *Beijerinckvirinae* and *Slopekvirinae* cliques. Additionally, Bolek and Lolek directly connect to one of the members of the *Slopekvirinae*—the *Shigella* phage HRP29 (NC_048174.1) (Table S13). The Clinker comparison showed that the Bolek and Lolek phages demonstrated similarity to *Beijerinckvirinae* phages through 10–14 of 57–61 proteins, respectively, and through 15 (of 57–61) to the *Shigella* phage HRP29 (Figure S16). These were some proteins involved in DNA replication and metabolism (helicase, ligase, polymerase, 5′-3′exonuclease, endonuclease VII), RNA polymerase, structural proteins (virion assembly, head–tail connector, scaffolding, major capsid, tail tubular A and B proteins), and terminase small and large subunits. Nonetheless, most of the proteins (18 of 57–61) of the Bolek and Lolek were shared with the *Aeromonas* virus PS, and in addition to those mentioned above, two proteins with unknown function and internal virion proteins B and C (Figure S16). The vConTACT VC classified both the Bolek and Lolek phages with the same overlap status, partially covering only one *Beijerinckvirinae*—the *Acinetobacter* phage vB_AbaP_Acibel007 [106] (Table S13). Based on the localization within the network and the phylogenetic trees obtained using RNA polymerase and major capsid proteins (Files S4 and S5), the Bolek, Lolek, and PS phages could be considered as members of the *Beijerinckvirinae* subfamily. According to the ratification of *Autographiviridae* and the thresholds of 50% and 95% of nucleotide sequence identity for genus and species determination, respectively [97], Bolek and Lolek presumably represent two distinct species (see above) of a novel genus (with the proposed name *Bolekvirus*).

It must also be noted that within the SG7 subgraph, there are also phages that are not yet classified into any subfamily (Figure 5; colored grey); however, based on their co-localization and vConTACT’s classification, most of them might be assigned to the subfamily of the phages clustered with them (Table S10). Among these subclusters, there is also another example of inter-family connections between phages. This includes autographiviruses (10 *Okabevirinae* phages and 9 phages yet unassigned to any subfamily but located within the *Okabevirinae* clique) and six siphoviruses infecting *Stenotrophomonas* (BUCT603, MW934263.1) and *Xanthomonas* hosts (phiL7 [107], CP1 [108], OP1 [109], Xp10 [110], and Xop411 [110]). Similarities are shared through the entire replication module (Figure S15), that is, their DNA primases, DNA helicases, DNA polymerases, DUF669-containing proteins, 5′-3′ exonucleases, DNA endonucleases VII, ribonuclease H-like domain-containing proteins RNA polymerases, and lysozymes (Figure S17). It is worth emphasizing that besides protein homology, the order of genes encoding them is also conserved and follows the canonical phiKMV-like gene order [111]. Moreover, phages OP1, Xop411, and Xp10 share DNA ligases with autographiviruses; however, the localization of genes encoding them is different (downstream RNAP gene) than in the genomes of phiKMV-like phages (upstream RNAP gene, Figure S17). The presence of the homologous replication region and lysozyme in both the sipho- and autographiviruses infecting the bacteria of the closely related genus of the *Xanthomonadaceae* family (*Stenotrophomonas, Xanthomonas, Xyella*) may be explained as a result of the adaptation of the replication and lysis processes to the similar host mechanisms and similar structure of the cell wall, respectively. However, the presence of the RNA polymerase itself in this set of homologous proteins is a very interesting issue as the occurrence of the RNAP gene in the genomes of *Siphoviridae* phages is very rare, while it is the hallmark of the *Autographiviridae* family. RNAP genes were also identified in two other siphoviruses infecting *Gammaproteobacteria*—the *Colwellia* phage 9A [112] and the *Shewanella* KASIA phage [26]. However, in this case, RNAP genes precede structural modules and are in the opposite orientation to the replication modules.

The presented analysis is congruent with the recent taxonomic ratification of autographiviruses [96] and previous comparative studies of the *Autographiviridae* family that focused mostly on phylogenetic analyses of individually characterized viruses in relation to their closest relatives based on the major capsid protein [113,114,115,116] terminase large subunit [93,113,115,116,117,118], RNA polymerase [91,114,116,117,118], DNA polymerase [23,114,116,119], tail tubular proteins [120], or head–tail connector [114] used as marker proteins, as well as with the analyses of the whole proteome of some groups of *Autographiviridae* representatives known at that time [22,84,121] using different databases (RefSeqv88, NCBI GenBank, and ACLAME, respectively). However, our global approach is the first to use a much larger number of phages (over 17,470 available in the INPHARED database) than was included in previous studies, adding recently discovered autographiviruses and those from metagenomic studies. Along with the support of phylogenetic analyses and insightful inspection of the similarities between the selected groups of phages, it provides broader insight into the relationships among the members of this family and the identification of their connections with representatives of other families. Finally, it also allows for updating the taxonomic position of 62 previously unclassified phages mainly because their potential taxonomic rank was under-sampled.

## 4. Conclusions

In this study, we described three novel virulent *Autographiviridae* phages—vB_AspA_Bolek, vB_AspA_Lolek, and vB_AspA_Tola—infecting different, yet closely related *Aeromonas* strains (MR7, MR19, and MR16, respectively) cohabitants of arsenic-containing microbial mats present in the Zloty Stok gold mine. To our best knowledge, these host–pathogen pairs are the first truly environmental *Aeromonas*-autographivirus systems that, it is worth emphasizing, coexist in the same community. The host ranges of the studied phages were restricted to individual strains from the gold mine without cross-infections—the host for one phage was a “nonhost” for the others. Bolek and Lolek seem particularly interesting as they are very similar to each other at the nucleotide level and share most of the proteins, except the highly diverged middle and C-terminal regions of their tail fibers. Thus, in the future, these phages can become a model system to investigate the properties of capsid proteins important for host range discrimination.

The studied phage–host systems were isolated from a constant low-temperature environment. The experimental analysis confirmed cold activity (i.e., efficient production of plaques at 4–25 °C) of all three phages and the cold tolerance of their host, albeit with a much wider temperature range of propagation (4–37 °C). This is another example from this gold mine of the phage–host system where bacteria seem to have greater flexibility to live in variable parameters of the environment than their parasites [26].

The nucleotide sequence analysis of the Bolek, Lolek, and Tola genomes revealed that they were significantly different from the viral genomes available in public databases, suggesting to recognize them as three novel species within *Autographiviridae*. Nonetheless, the protein-based network, together with phylogenetic analysis, allowed for a refinement of their taxonomic classification. Hereby, Bolek and Lolek might be assigned to two novel species within the proposed novel genus (*Bolekvirus*) within the *Beijerinckvirinae* subfamily, while Tola could be classified as a member of a new species in the *Gajwadongvirus* genus within the *Colwellvirinae* subfamily.

Moreover, a protein-based similarity network showed that 27 *Autographiviridae* members infecting *Aeromonas* were scattered across five different subgraphs, but most of them (17 belonging to the *Melnykvirinae* subfamily) formed one dense cluster (SG4, Figure 5). The 10 others were significantly and mutually distinct. Bolek, Lolek, and Tola are among this group of “outliers”, which further emphasizes their uniqueness.

INPHARED database resources, including twice as many *Autographiviride* genomes (944) as that in the last ICTV proposal (471 in 2019), were used by us for comparative genomic analysis of all known members of this family and their relatives. The obtained results were in agreement with the ICTV classification. However, it is worth noting that this protein-based network grouped many unclassified representatives together with presumably related members of various *Autographiviridae* subfamilies, which may facilitate their future taxonomic classification. Moreover, this analysis brought up intriguing relationships of the *Autographiviridae* phages with 28 representatives of other *Caudoviricetes* families, particularly between the autographiviruses of the *Okabevirinae* subfamily and six *Siphoviridae* phages sharing similarities and synteny of the whole DNA replication region and, most importantly, an RNAP polymerase gene—the hallmark of the *Autographiviridae*.

## Figures and Tables

**Table 1:** Characteristics of *Aeromonas* sp. MR7, MR16, and MR19 genomes.

	MR7	MR16	MR19
Number of contigs	54	97	61
Sequenced genome size (bp)	4,822,910	4,655,773	4,883,405
GC content (%)	60.4	60.9	60.2
Number of predicted genes	4364	4236	4367
Number of putative virulence factors Antibiotic Resistance/Virulence Factor	56/59	41/49	51/49
Number of prophages	1	3	1
Number of genes encoding transposases	14	52	22

**Table 2:** Comparison of latency times, rise periods, and burst sizes of the Bolek, Lolek, and Tola phages in LB and R2A media at 20 °C.

	Bolek		Lolek		Tola	
	R2A	LB	R2A	LB	R2A	LB
Latency period (min)	60	60	60	60	70	60
Rise period (min)	70	70	20	20	20	30
Burst size (PFU/IC)	73 ± 8	70 ± 10	68 ± 8	62 ± 16	107 ± 8	115 ± 10

**Table 3:** Characteristics of the Bolek, Lolek, and Tola genomes.

	Bolek	Lolek	Tola
Sequenced genome size (bp)	42,433	43,136	45,276
Physical genome size (bp)	42,747	43,638	45,907
Terminal repeats size (bp)	314	502	631
GC content (%)	52.7	52.6	47.4
Number of predicted genes	57	61	70
Number of predicted gene products	27	27	32
Number of hypothetical proteins	30	34	38

## Supplementary Materials

The following are available online at https://www.mdpi.com/article/10.3390/v14051016/s1, Figure S1: The comparison of the *Aeromonas* sp. MR7, MR16, MR19, and O23A genomes generated by OrthoVenn2 tool; Figure S2: Growth of the *Aeromonas* sp. MR7, MR16, and MR19 strains at (a) 10 °C and (b) 20 °C, in liquid LB and R2A media; Figure S3: Visualization of the Bolek, Lolek, and Tola plaques obtained on the LB medium; Figure S4: Visualization of Bolek plaques surrounded by an opaque-looking halo zone; Figure S5: Genome size distribution of *Autographiviridae* phages; Figure S6: Comparison of the putative Bolek, Lolek, and Tola tail fiber proteins; Figure S7: The alignment of the Tola and SP6 genomes; Figure S8: Comparison of the Bolek and Lolek genomes generated by EasyFig; Figure S9: Protein-based phage similarity network; Figure S10: Protein-based phage similarity network of Figure 5 where all phages mentioned in the text are labelled with names; Figure S11: The alignment of the *Zobellviridae* phages and their neighborhood of SG3; Figure S12: The alignment of the *Podoviridae* phages and their neighborhood of SG3; Figure S13: The genome alignment of the siphovirus Kolga and their neighborhood of SG3; Figure S14: The genome alignment of the *Siphoviridae* phages and their neighborhood of SG3; Figure S15: The genome alignment of the Tola phage and its neighborhood of SG5; Figure S16: The alignment of the Bolek and Lolek and their selected neighborhood of SG7; Figure S17: The alignment of the *Siphoviridae* phages and their neighborhood of SG7; Table S1: Characteristics of annotated *Autographiviridaephage*, infecting *Aeromonas*, whose genomes are available in the NCBI Viral RefSeq database; Table S2: List of strains used in this study; Table S3: Antibiotic resistance and virulence genes of *Aeromonas* sp. MR7, MR16, and MR19 strains; Table S4: Defense systems identified with the use of the Prokaryotic Antiviral Defense LOCator (PADLOC) in the *Aeromonas* MR7, MR16, and MR19 genomes; Table S5: Appearance of single colonies of *Aeromonas* sp. MR7, MR16, and MR19 strains after incubation of plates at various temperatures (4–42 °C) in LB and R2A media; Table S6: Adsorption efficiencies of the Bolek, Lolek, and Tola phages to the MR7, MR16, and MR19 cell; Table S7: Genes located within the vB_AspA_Bolek phage; Table S8: Genes located within the vB_AspA_Lolek phage; Table S9: Genes located within the vB_AspA_Tola phage; Table S10: Phages of the SG1, SG2, and SG7 subgraphs unclassified yet to any subfamily with the proposed assignment to the subfamily rank based on the vConTACT analysis; Table S11: vConTACT-based neighbors of the vB_AspA_Tola phage; Table S12: vConTACT-based members of the subgraph SG6; Table S13: vConTACT-based neighbors of the vB_AspA_Bolek and Lolek phages; File S1: The vB_AspA_Bolek PhageTerm Analysis report; File S2: The vB_AspA_Lolek PhageTerm Analysis report; File S3: The vB_AspA_Tola PhageTerm Analysis report; File S4: Phylogenetic tree based on the RNA polymerase gene; File S5: Phylogenetic tree based on the major capsid protein gene.
