# Supplementary material for: Characterization of Three Novel Virulent Aeromonas Phages Provides Insights into the Diversity of the Autographiviridae Family
Source: Viruses. 2022 May 10;14(5):1016. doi: 10.3390/v14051016 (PMC9145550; doi:10.3390/v14051016)
Supplement: Supplementary file 1 [file viruses-14-01016-s001.zip › viruses-1673303-supplementary/SUPPLEMENTARY/Supplementary_Figures.pdf]

Supplementary materials for

Characterisation of Three Novel Virulent *Aeromonas* Phages Provides Insights into the Diversity of the *Autographiviridae* family

Katarzyna Bujak, Przemyslaw Decewicz, Michal Kitowicz and Monika Radlinska *

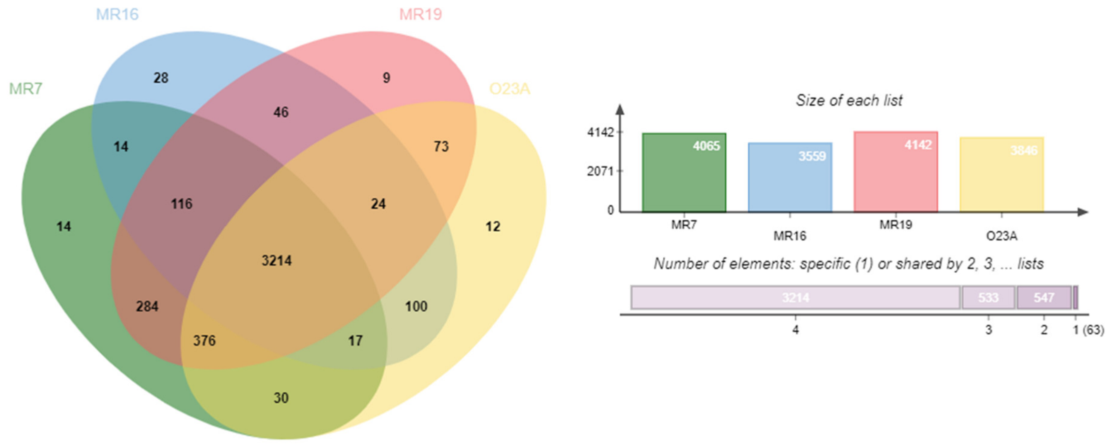

Figure S1. The comparison of the *Aeromonas* sp. MR7, MR16, MR19, and O23A genomes generated by OrthoVenn2 tool.

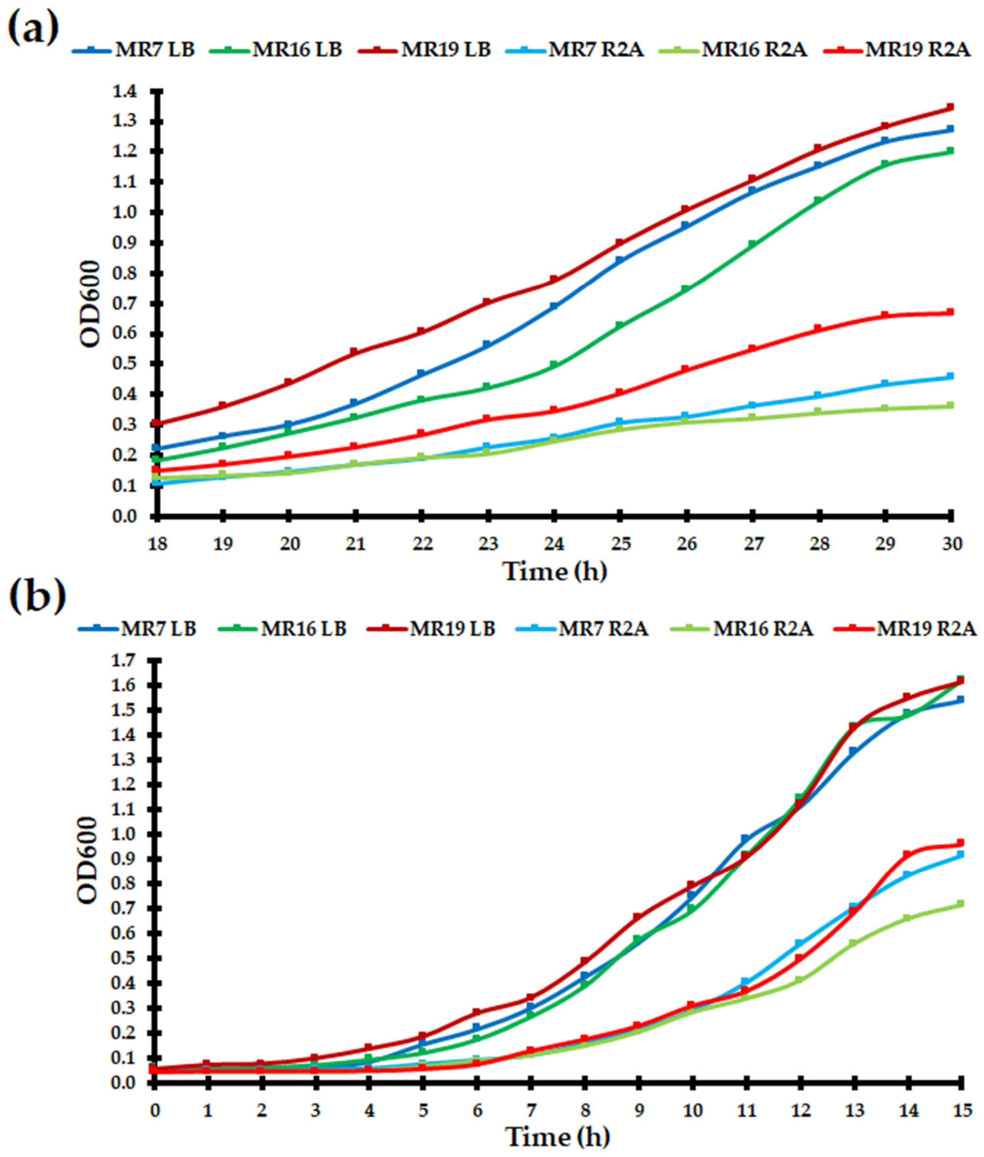

Figure S2. Growth of the *Aeromonas* sp. MR7, MR16, and MR19 strains at **(a)** 10 °C and **(b)** 20 °C, in liquid LB and R2A medium.

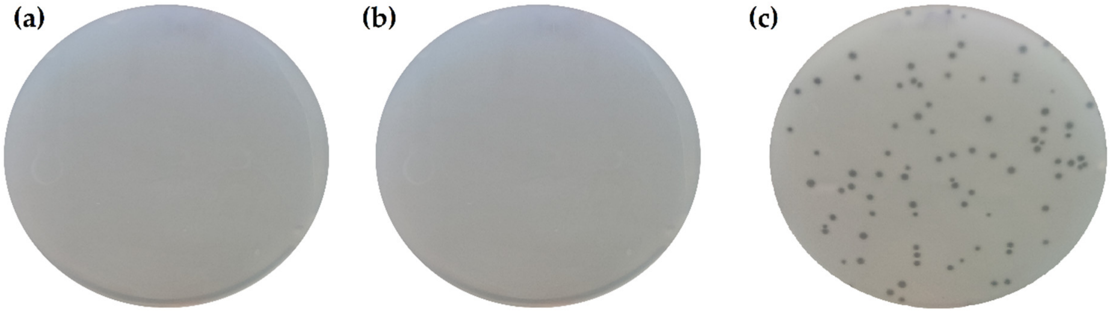

Figure S3. Visualisation of the Bolek, Lolek and Tola plaques obtained on the LB medium.

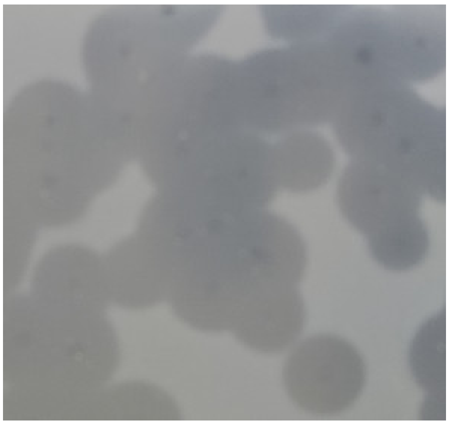

Figure S4. Visualization of Bolek plaques surrounded by an opaque-looking halo zone.

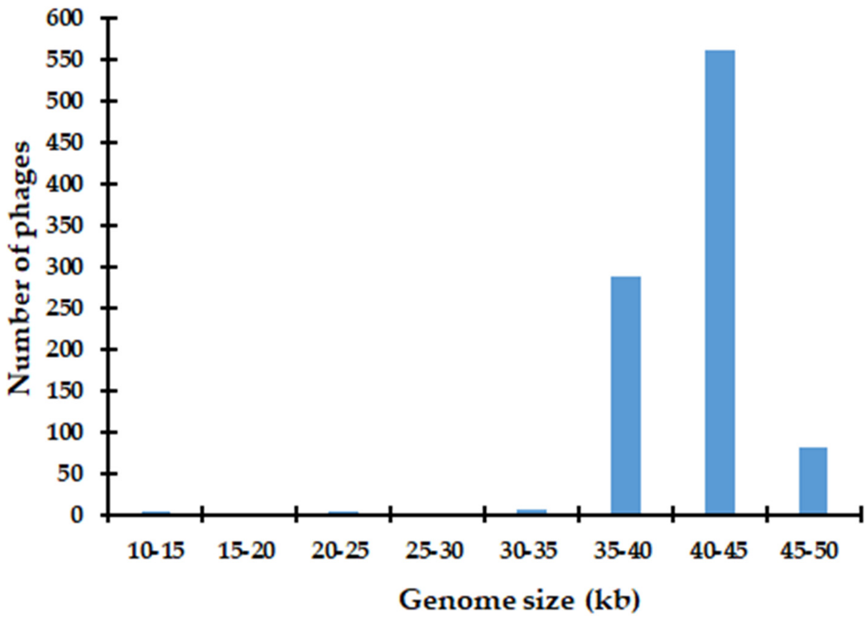

Figure S5. Genome size distribution of *Autographiviridae* phages.

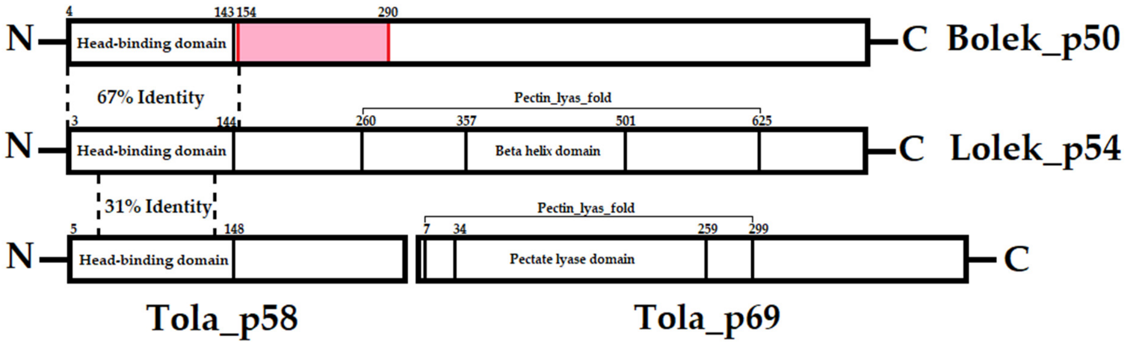

Figure S6. Comparison of the putative Bolek, Lolek and Tola tail fibre proteins. Domains and superfamilies identified by InterPro tool are marked in black. Putative region responsible for depolymerase activity identified by HHpred is marked in red.

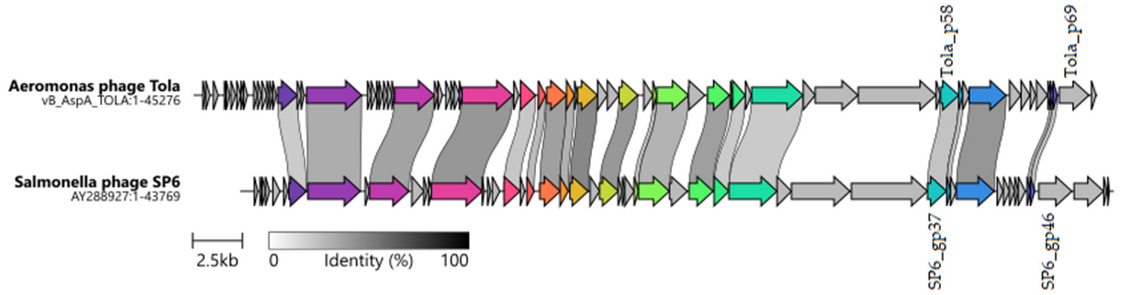

Figure S7. The alignment of the Tola and SP6 genomes. Each protein-encoding feature is presented as an arrow. The two arrows connected with a white-to-black coloured block correspond to their protein sequence identity level.

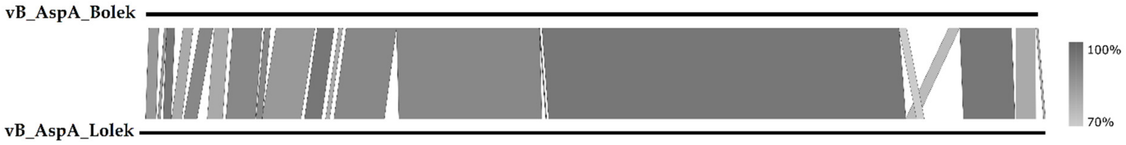

Figure S8. Comparison of the Bolek and Lolek genomes generated by EasyFig.

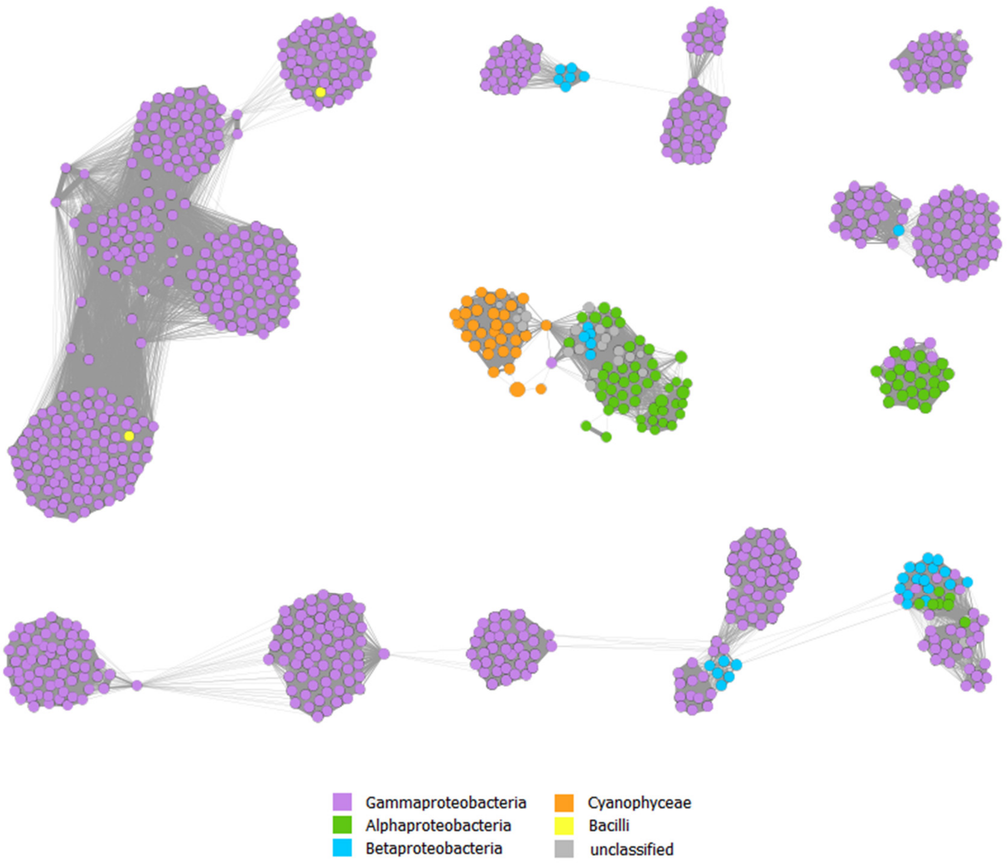

Figure S9. Protein-based phage similarity network. Only *Autographiviridae* phages and phages showing significant similarity reflected by edges between them are shown. The colouring of the nodes is based on the current taxonomic classification of the bacteria, i.e. the class rank of the phage hosts. The edge thickness reflects the degree of similarity between two phages (the greater the similarity, the thicker the edge), considered as an overall similarity of proteins encoded by a pair of phages.

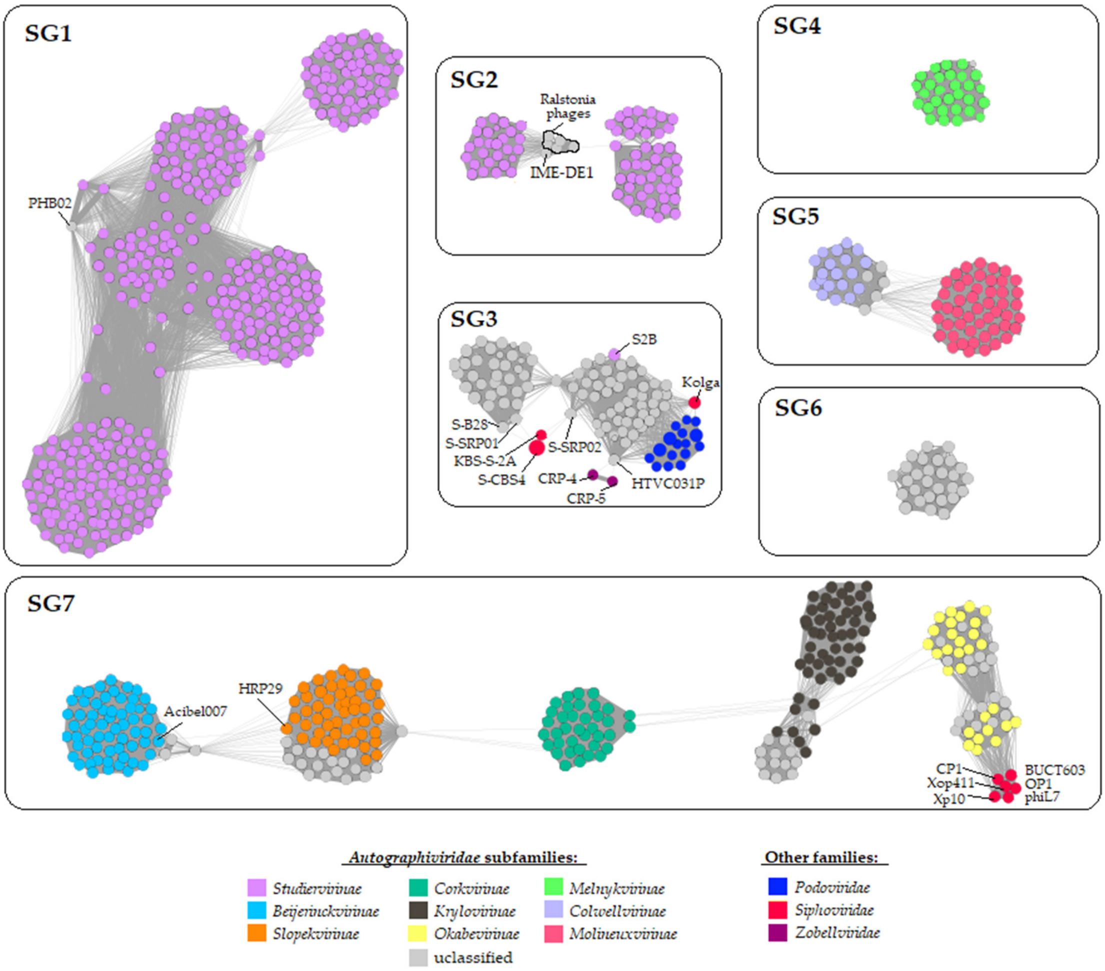

Figure S10. Protein-based phage similarity network of Figure 5 where all phages mentioned in the text are labelled with names.

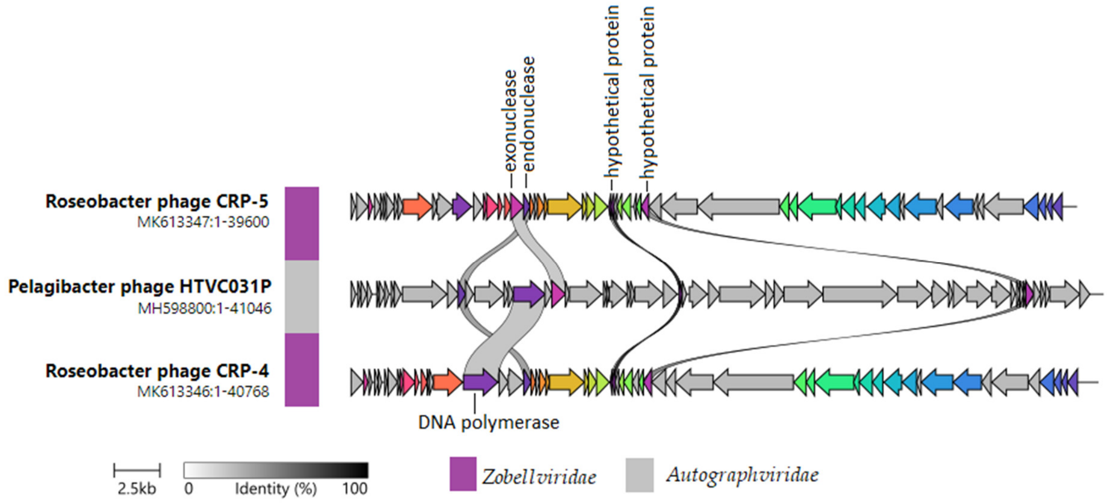

Figure S11. The alignment of the *Zobellviridae* phages and their neighbourhood of SG3. Phage genomes are delineated and reversed if required. Each protein-encoding feature is presented as an arrow. The two arrows connected with a white-to-black coloured block correspond to their protein sequence identity level.

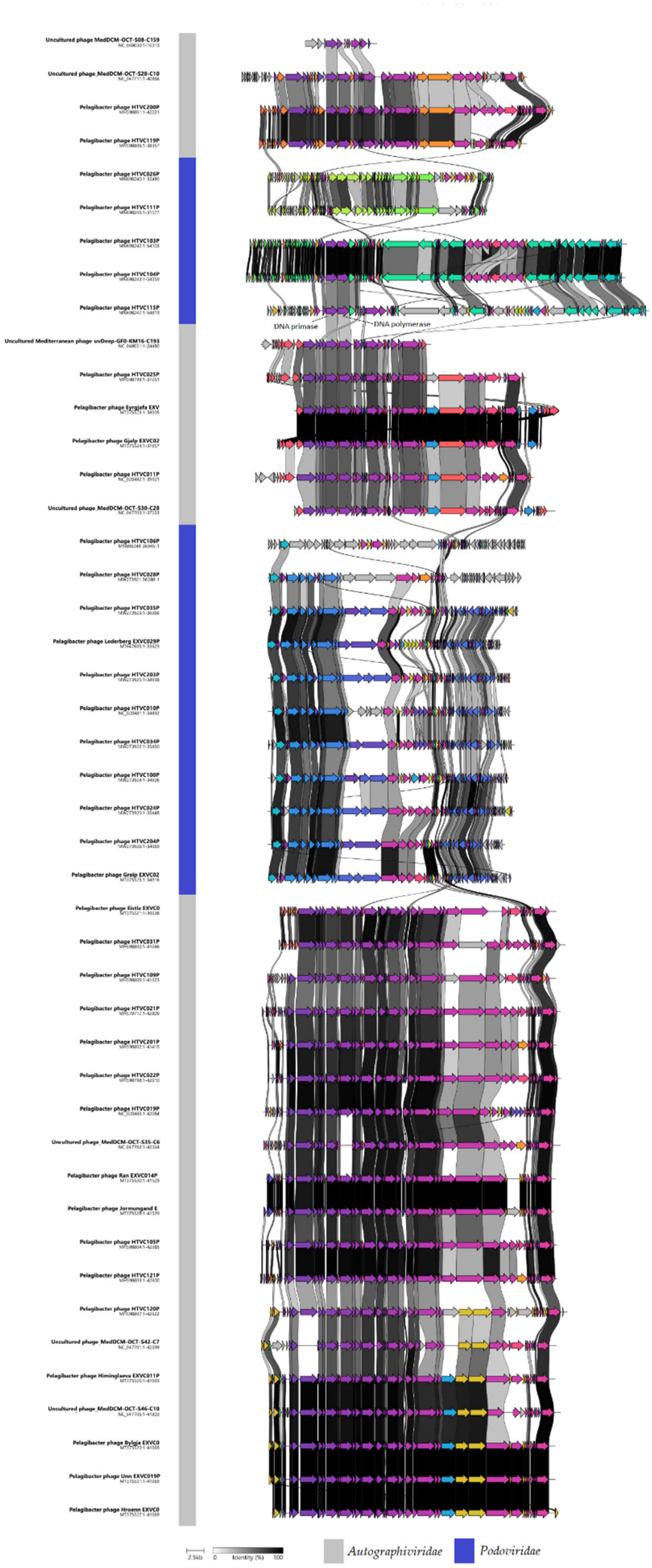

Figure S12. The alignment of the *Podoviridae* phages and their neighbourhood of SG3. Phage genomes are delineated and reversed if required. Each protein-encoding feature is presented as an arrow. The two arrows connected with a white-to-black coloured block correspond to their protein sequence identity level.

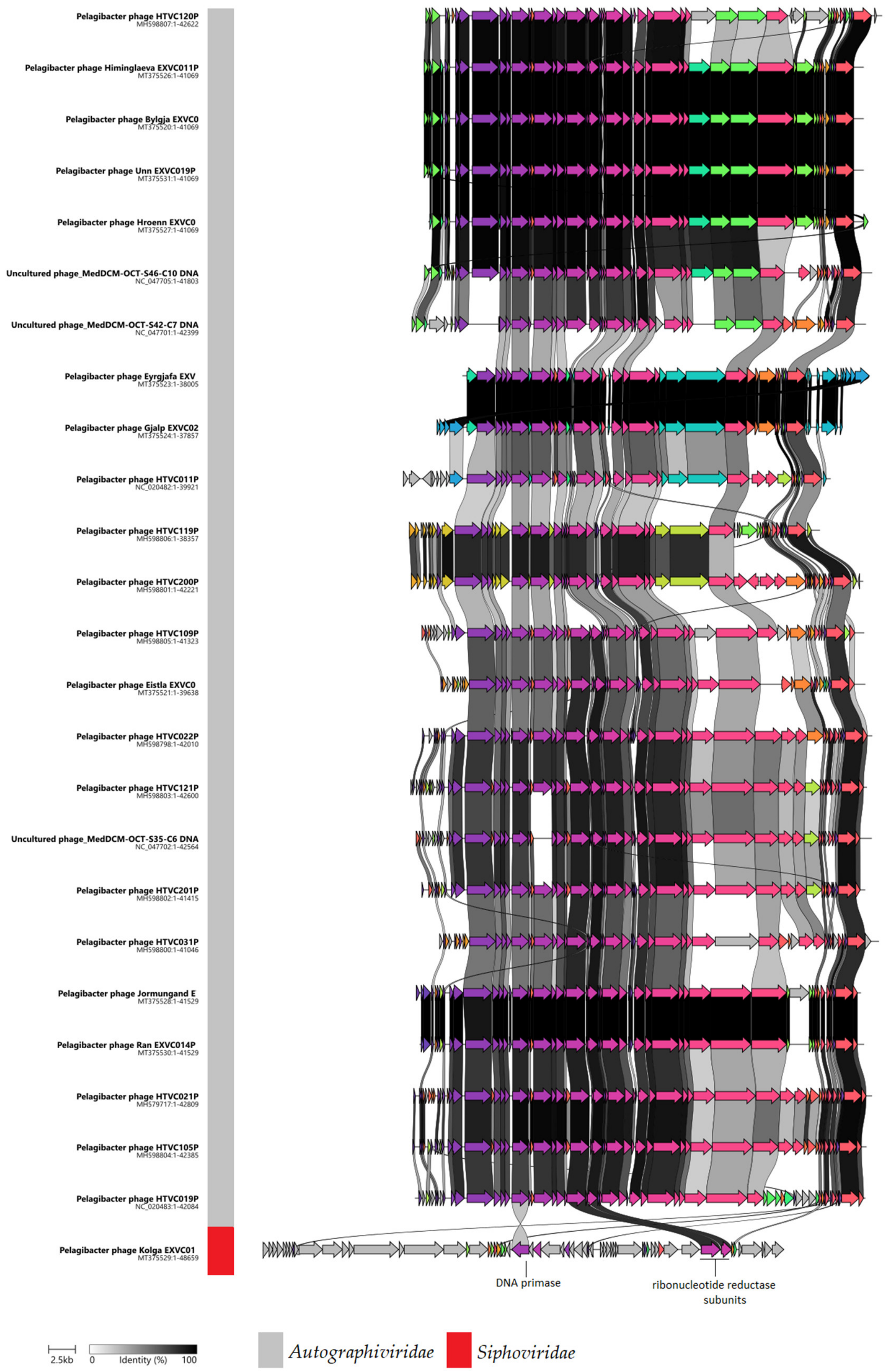

Figure S13. The genome alignment of the siphovirus Kolga and their neighbourhood of SG3. Phage genomes are delineated and reversed if required. Each protein-encoding feature is presented as an arrow. The two arrows connected with a white-to-black coloured block correspond to their protein sequence identity level.

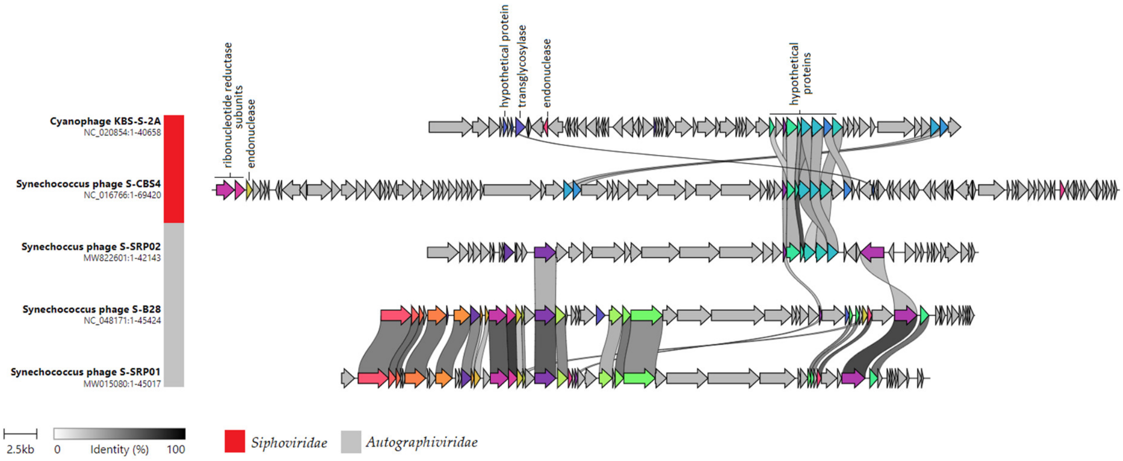

Figure S14. The genome alignment of the *Siphoviridae* phages and their neighbourhood of SG3. Phage genomes are delineated and reversed if required. Each protein-encoding feature is presented as an arrow. The two arrows connected with a white-to-black coloured block correspond to their protein sequence identity level.

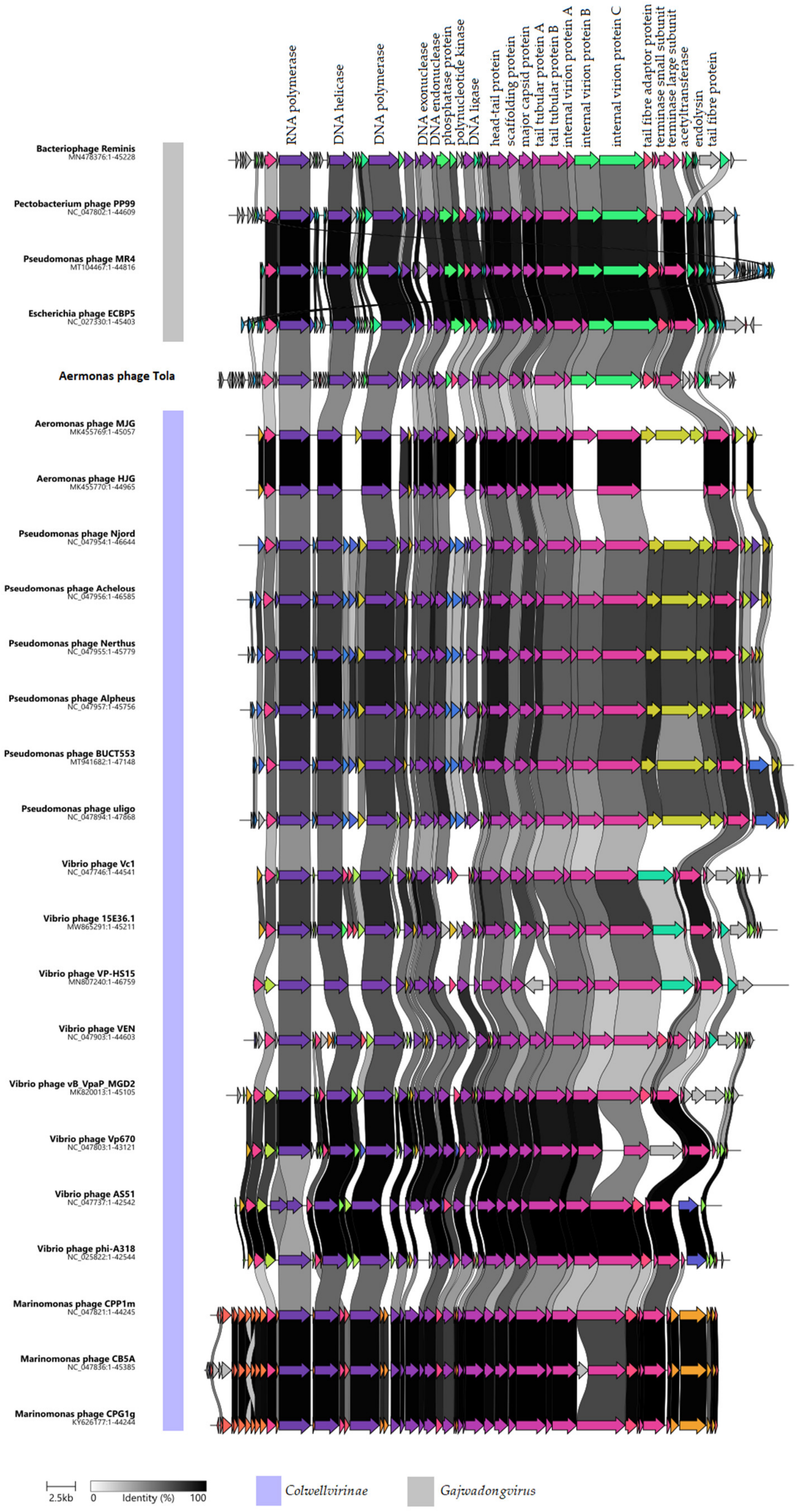

Figure S15. The genome alignment of the Tola phage and its neighbourhood of SG5. Phage genomes are delineated and reversed if required. Each protein-encoding feature is presented as an arrow. The two arrows connected with a white-to-black coloured block correspond to their protein sequence identity level.

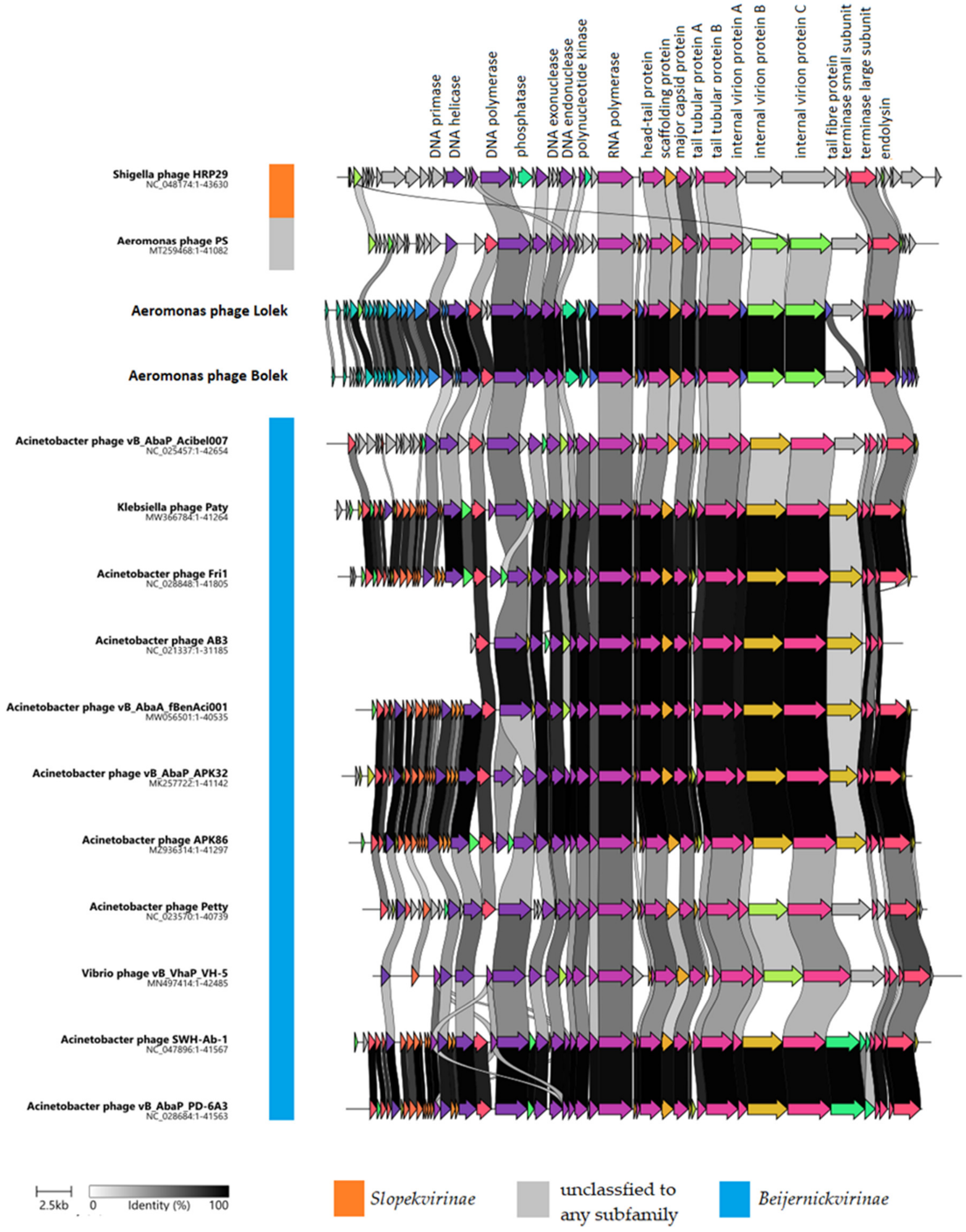

Figure S16. The alignment of the Bolek, Lolek and their selected neighbourhood of SG7. The group of 43 *Acinetobacter* phages very similar to phage Fri1 was not shown for better clarity of figure. Phage genomes are delineated and reversed if required. Each protein-encoding feature is presented as an arrow. The two arrows connected with a white-to-black coloured block correspond to their protein sequence identity level.

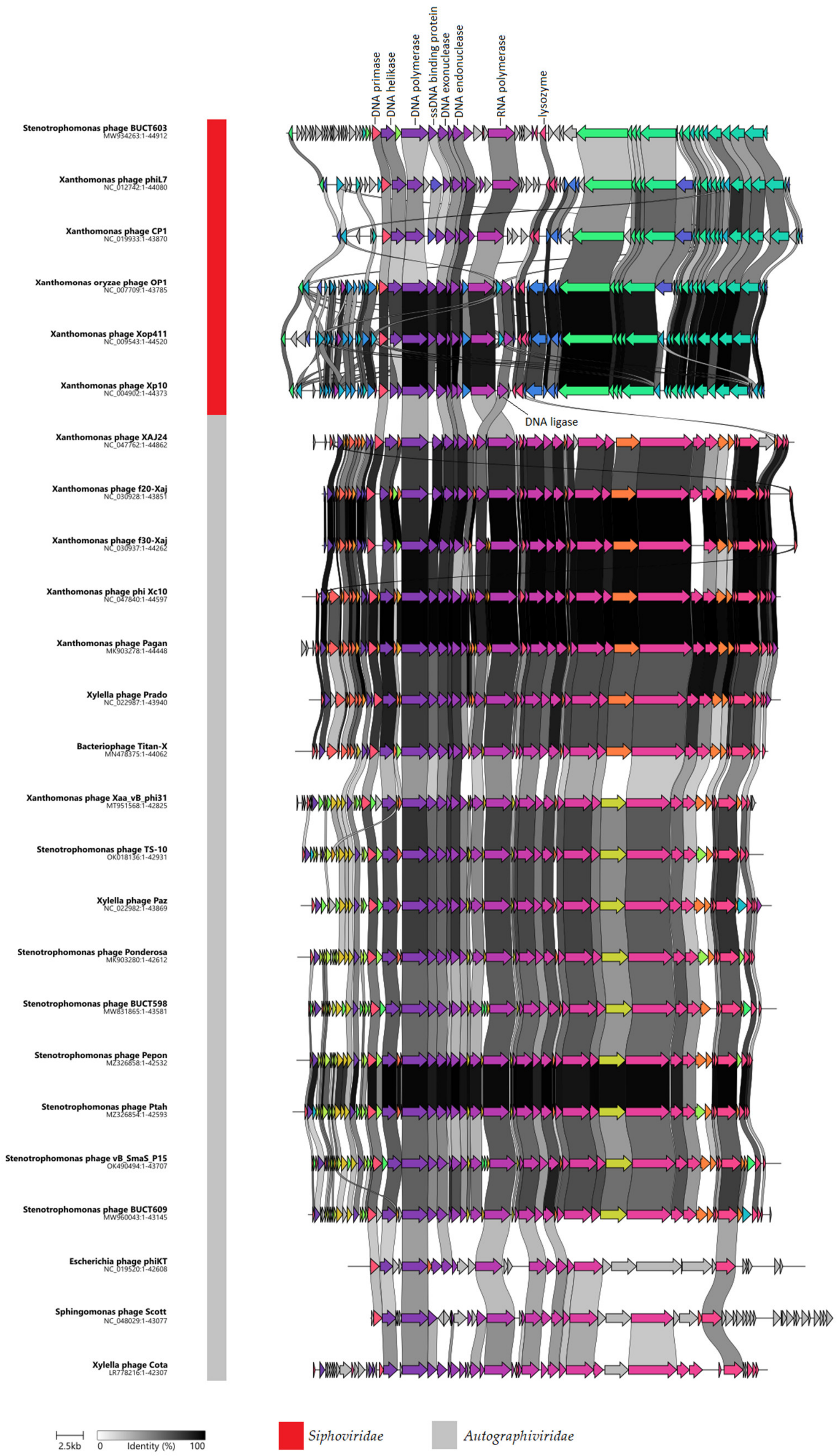

Figure S17. The alignment of the *Siphoviridae* phages and their neighbourhood of SG7. Phage genomes are delineated and reversed if required. Each protein-encoding feature is presented as an arrow. The two arrows connected with a white-to-black coloured block correspond to their protein sequence identity level.
