# Supplementary figures and images for: Characterization of Three Novel Virulent Aeromonas Phages Provides Insights into the Diversity of the Autographiviridae Family
Source: Viruses. 2022 May 10;14(5):1016. doi: 10.3390/v14051016 (PMC9145550; doi:10.3390/v14051016)

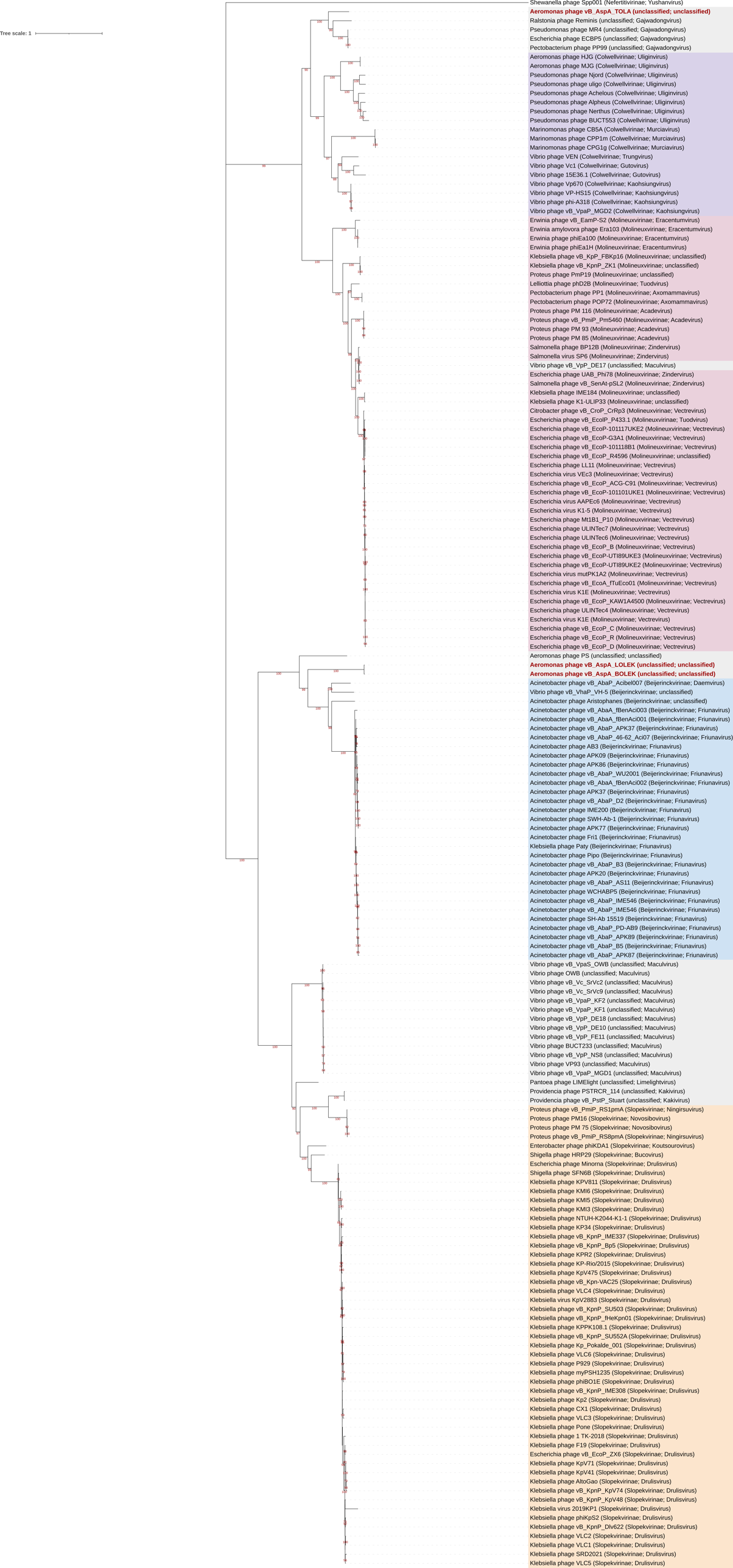

Supplement: Supplementary file 1 [file viruses-14-01016-s001.zip › viruses-1673303-supplementary/SUPPLEMENTARY/Supplementary_Files/File_S4_Phylogenetic_tree_based_on_the_RNA_polymerase_gene.png]
